# Entorhinal Layer II Calbindin-Expressing Neurons Originate Widespread Telencephalic and Intrinsic Projections

**DOI:** 10.3389/fnsys.2019.00054

**Published:** 2019-10-15

**Authors:** Shinya Ohara, Michele Gianatti, Kazuki Itou, Christin H. Berndtsson, Thanh P. Doan, Takuma Kitanishi, Kenji Mizuseki, Toshio Iijima, Ken-Ichiro Tsutsui, Menno P. Witter

**Affiliations:** ^1^Kavli Institute for Systems Neuroscience, Center for Computational Neuroscience, Egil and Pauline Braathen and Fred Kavli Centre for Cortical Microcircuits, Norwegian University of Science and Technology (NTNU), Trondheim, Norway; ^2^Laboratory of Systems Neuroscience, Graduate School of Life Sciences, Tohoku University, Sendai, Japan; ^3^Department of Physiology, Graduate School of Medicine, Osaka City University, Osaka, Japan

**Keywords:** medial entorhinal cortex, lateral entorhinal cortex, parahippocampus, connectivity, rodent, commissural projections, long-range intrinsic projections

## Abstract

In the present study we provide the first systematic and quantitative hodological study of the calbindin-expressing (CB+) principal neurons in layer II of the entorhinal cortex and compared the respective projections of the lateral and medial subdivisions of the entorhinal cortex. Using elaborate quantitative retrograde tracing, complemented by anterograde tracing, we report that the layer II CB+ population comprises neurons with diverse, mainly excitatory projections. At least half of them originate local intrinsic and commissural projections which distribute mainly to layer I and II. We further show that long-range CB+ projections from the two entorhinal subdivisions differ substantially in that MEC projections mainly target field CA1 of the hippocampus, whereas LEC CB+ projections distribute much more widely to a substantial number of known forebrain targets. This connectional difference between the CB+ populations in LEC and MEC is reminiscent of the overall projection pattern of the two entorhinal subdivisions.

## Introduction

The entorhinal cortex (EC) is conceived as the nodal point in the cortico-hippocampal network that is critically involved in memory and spatial navigation (Schenk and Morris, 1985; Brun et al., 2002, 2008; Eichenbaum et al., 2007; Ji and Maren, 2008; Reagh and Yassa, 2014; Rodo et al., 2016). Anatomically, EC can be divided into two functionally distinct subdivisions, lateral and medial EC (LEC and MEC, respectively). A substantial proportion of neurons in MEC are spatially modulated, reflecting self-location relative to the geometry of the environment. In contrast, in LEC such spatial modulation is essentially absent, with activity correlating to odors or objects in context (Fyhn et al., 2004; Deshmukh and Knierim, 2011; Neunuebel et al., 2013; Tsao et al., 2013; Moser et al., 2014) or reflecting the temporal progression of the experimental event (Tsao et al., 2018; Montchal et al., 2019).

We previously showed that differences in morphological and physiological properties exist between MEC and LEC in layer II neurons, whereas differences in other layers are not evident (Canto and Witter, 2012a, b; Cappaert et al., 2015). Principal cells in EC layer II come in two chemical types, calbindin- and reelin-expressing cells (CB+ and RE+, respectively), and interestingly, these two neuron-types distribute differently in the two subdivisions. In the rodent MEC, the two types appear to be grouped in patches, while in LEC they form two separate sublayers, RE+ cells superficially (IIa) and CB+ cells deeper (IIb) (Tuñón et al., 1992; Fujimaru and Kosaka, 1996; Wouterlood, 2002; Ramos-Moreno et al., 2006; Kitamura et al., 2014; Ray et al., 2014; Leitner et al., 2016). Taken together, these data indicate that layer II principal neurons may contribute to the phenotypical differences between MEC and LEC. However, studies on the local networks of RE+ neurons show a striking similarity between LEC and MEC (Pastoll et al., 2012; Couey et al., 2013; Fuchs et al., 2016; Leitner et al., 2016; Nilssen et al., 2018), and in both subdivisions RE+ cells are the exclusive origin of the projections to dentate gyrus, and hippocampal fields CA2 and CA3 (Varga et al., 2010; Ray et al., 2014; Witter et al., 2017). Therefore, a difference in the connectional organization of CB+ layer II neurons might be relevant to explore.

Recent studies have proposed that MEC CB+ pyramidal cells play an important role in generating grid cell activity, which was related to their anatomical clustering, rhythmicity, cholinergic modulation (Ray et al., 2014), and spatial discharge properties (Tang et al., 2014). On the other hand, LEC CB+ pyramidal cells are proposed to have a functional role in top-down modulation of olfactory processing (Leitner et al., 2016).

The projections of CB+ neurons in MEC and LEC have only been described in incidental reports. In case of MEC CB+ neurons, projections to the hippocampus (Wouterlood, 2002), more specifically to stratum lacunosum of CA1 (Kitamura et al., 2014), to contralateral MEC (Varga et al., 2010), ipsilateral MEC (Zutshi et al., 2018), and the medial septum (MS) (Fuchs et al., 2016) have been described. For LEC CB+ neurons, projections to CA1 (Kitamura et al., 2014), to contralateral LEC, the olfactory bulb, and piriform cortex (Leitner et al., 2016) have been reported. Although these previous studies described the targets of the CB+ neurons, the proportion of the CB+ neurons contributing to each of these projections was not provided. Hence, it is unclear whether all CB+ neurons or only part of them project to the target regions. Furthermore, a systematic and quantitative comparison of the efferent connectivity of CB+ populations in LEC and MEC is lacking. In the present study, we used combinations of quantitative retrograde tracing and immunohistochemical approaches, supplemented with anterograde tracing, to assess projections of CB+ neurons in layer II of both MEC and LEC in rats. Our analysis included all known major EC projections and showed that the CB+ population in layer II is composed of diverse neurons having distinct projections. Most importantly, layer II CB+ neurons in both entorhinal subdivisions are a main source of an elaborate local excitatory projection within EC. We further demonstrated LEC CB+ neurons mediate widespread forebrain projections, whereas the projections of MEC CB+ neurons distribute axons almost exclusively within the hippocampus and the EC.

## Materials and Methods

### Surgical Procedures and Tracer/Virus Injections

Either adult male Wistar rats weighing 200–230 g, adult female Sprague Dawley rats weighing 230–285 g, or adult female Long Evans rats 210–280 g were used in this study. All experiments using Wistar rats were performed at Tohoku University. The experiments were approved by the Center for Laboratory Animal Research, Tohoku University, and were conducted according to the Guidelines of the National Institutes of Health and the Tohoku University Guidelines for Animal Care and Use. All experiments using Sprague Dawley rats, Long Evans rats, and GAD67 transgenic mice expressing GFP (Tanaka et al., 2003) were performed at the Kavli Institute for Systems Neuroscience/Centre for Neural Computation at the Norwegian University of Science and Technology (NTNU), where animals were housed and handled according to the Norwegian laws and regulations concerning animal welfare and animal research. Experimental protocols were approved by the Norwegian Animal Research Authority and were in accordance with the European Convention for the Protection of Vertebrate Animals used for Experimental and Other Scientific Purposes.

Under deep anesthesia either with isoflurane or with ketamine (80.0 mg/kg, i.p.) and xylazine (0.8 mg/kg, i.p.), rats were mounted in a stereotaxic frame. The skull was exposed, and a small burr hole was drilled above the injection site. Retrograde tracers were injected into the target areas by pressure injection using a glass micropipette (tip diameter = 20–40 μm) either connected to a 1 μl Hamilton microsyringe or to an automated microinjection pump (WPI Nanoliter, 2010). Three fluorescent retrograde tracers were used in rats in the following parameter: 50–200 nl of fluorogold (FG; 2.5% in H_2_O, Fluorochrome), 100–500 nl of Alexa Fluor 555 conjugated Cholera Toxin Subunit B (CTB-555; 1 mg/ml in phosphate-buffered saline (PBS), Thermo Fisher), 100 nl of Fast Blue (FB; 1% in PBS, EMS-Grivory). The coordinates of the injection sites and detailed information of each sample are shown in Supplementary Table 1. Some samples were also used in our previous study (Ohara et al., 2018). For retrograde tracing experiments in mice, red retrobeads (Lumafluor) and Fast Blue were used. After the injection, at 25 nl per minute, the pipette was left in place for another 15 minutes before it was withdrawn. The wound was sutured, and the animal was monitored for recovery from anesthesia before being returned to its home cage. The survival periods were 5–7 days for these retrograde tracing experiments.

For dual anterograde tracing experiments, 2.5% *Phaseolus vulgaris*-leucoagglutinin (PHA-L; Vector Laboratories) and 3.5% 10 kDa biotinylated dextran amine (BDA, Invitrogen, Molecular Probes) were injected iontophoretically with positive 5 μA current pulses (6 s on; 6 s off) for 15 min in the following coordinates (anterior to either bregma (APb) or transverse sinus (APt), lateral to sagittal sinus (ML), ventral to dura (DV) in mm): MEC (layer II; APt + 0.5; ML 4.9, DV 2.9, angle 11 degrees in the sagittal plane with the glass micropipette pointing to rostral); MEC (layer III; APt + 1.0, ML 4.9, DV 2.9, angle 11 degrees in the sagittal plane with the glass micropipette pointing to rostral); LEC (layer II; APb -6.0, ML 6.8, DV 4.7); LEC (layer III; APb -8.3, ML 6.0, DV 4.0). The survival period for this anterograde tracing was 7 days.

For AAV double infection approach, 500 nl of retrograde-infecting AAV expressing Cre recombinase (AAV6-Cre; Aronoff et al., 2010) was injected into the border of LEC and MEC (APb -8.3, ML 6.0, DV 3.8) while 500 nl of Cre-dependent reporter AAV that expresses EYFP after recombination (AAV1/2-EF1α-DIO-EYFP) was injected into the rostral LEC (APb 6.0, ML 6.8, DV 4.7). The survival period was 3 weeks.

### Immunohistochemistry and Analysis

Following an appropriate survival period for each experiment, the animals were deeply anaesthetized with sodium pentobarbital (100 mg/kg, i.p.) and perfused transcardially either with 10% sucrose in 0.1 M phosphate-buffer (PB) or with Ringer’s solution (0.85% NaCl, 0.025% KCl, 0.02% NaHCO3) followed by 4% paraformaldehyde in 0.1M PB. The brains were removed from the skulls, postfixed in 4% paraformaldehyde in 0.1 M PB for 4 h at 4°C, and then cryoprotected either in PB containing 30% sucrose or in a mixture of 20% glycerol and 2% dimethyl sulfoxide (DMSO) for at least 48 h at 4°C. The brains were cut into 40–60 μm sections in either the coronal or horizontal plane on a freezing microtome.

For immunofluorescence staining, floating sections were washed in PBS, permeabilized with PBS containing 5% normal goat serum and 0.1% Triton-X 100 for an hour at room temperature, and then incubated overnight at 4°C with a rabbit anti-calbindin antibody (1:1000; Abcam), a rabbit anti-calbindin antibody (1:5000; Swant), or a mouse anti-reelin antibody (1:1000; Millipore) diluted in PBS containing 5% normal goat serum and 0.1% Triton-X 100. They were then washed PBS containing 0.1% Triton X-100 (PBT) and incubated for 2–6 h at room temperature in Cy5-conjugated goat anti-rabbit IgG (1:400; Jackson ImmunoResearch), Alexa 546-conjugated goat anti-rabbit IgG (1:800; Invitrogen Ltd.), or Alexa488-conjugated goat anti-mouse IgG (1:800; Invitrogen Ltd.) diluted in PBT. The sections were counterstained with either Hoechst 33258 (1:1000; Dojindo) or NeuroTrace 500/525 Green Fluorescent Nissl Stain (1:300; Invitrogen Ltd.), mounted on gelatin-coated slides, and coverslipped using Entellan new (Millipore). The brain sections were examined under a Zeiss Axiovert 200M microscope, and images were captured using an AxioCam MRm digital camera and Axiovision image processing software (Carl Zeiss). Degital images were also obtained using an automated scanner (Zeiss Axio Scan Z1). In order to precisely identify the location of the injection site in horizontally sectioned samples, we used the Waxholm space three-plane architectonic atlas of the rat hippocampal region (Papp et al., 2014; Boccara et al., 2015; Kjonigsen et al., 2015), and identified the corresponding location of the injection site in the coronal plane.

To quantify the colocalization of calbindin immunolabeling and retrograde labeling, confocal images of retrogradely labeled and immunohistochemically stained entorhinal neurons were acquired in sections taken at every 240 μm throughout EC, using a confocal microscope (LSM 5 Exciter and LSM 880, Carl Zeiss) with a 40× oil objective (Plan Apochromat 40× NA1.3 Oil, Carl Zeiss, Supplementary Figure 1). Since the signal of Calbindin immunolabeling decreases in the center of the sections in samples cut at a thickness of 60 μm, the confocal images were taken at the upper surface and lower surface of each section. We set the region of interest (ROI) as the area where there were a certain number of retrogradely labeled neurons in EC layer II, and quantified the number of retrogradely labeled neurons and immunohistochemically stained neurons in this ROI using ImageJ software^1^. Similar to previous studies (Varga et al., 2010; Kitamura et al., 2014; Fuchs et al., 2016; Leitner et al., 2016), the projection of CB+ neurons were analyzed by quantifying the percentage of double-labeled neurons among the retrogradely labeled neurons in layer II of MEC and LEC. This provided information of whether the projection to the targeted regions specifically originated from CB+ neurons. In addition, we examined the percentage of double-labeled neurons with respect to the total CB+ neuron population to examine the proportion of CB+ neurons that contributed to the targeted projections.

The data are shown as mean ± standard erros. Prism software was used for data analysis (Graphpad software), and the Wilcoxon signed rank test was used for the analysis of the hippocampal injection experiments. Friedman test followed with Dunn’s multiple comparisons post-test was used to compare groups in case of the entorhinal injection experiments.

## Results

### Distribution of Calbindin Neurons in LEC and MEC

**FIGURE 1:**
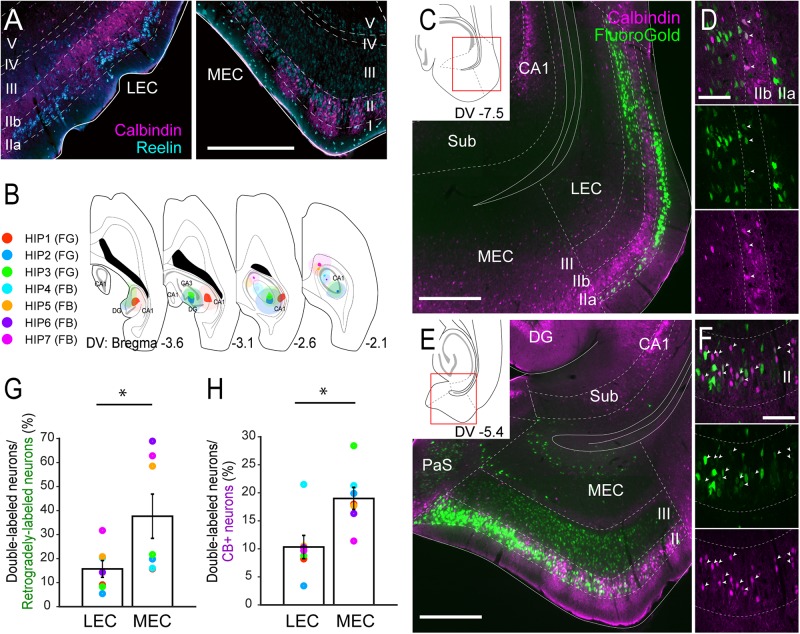
Projections of CB+ neurons in LEC and MEC to the hippocampus. **(A)** Distribution of neurons immunoreactive for RE (cyan) and CB (magenta) in rat LEC and MEC. **(B)** The injection sites of retrograde tracers, either FG or FB, in samples with hippocampal injection. Each injection is illustrated with a different color. For all cases, the dark color shows the injection site whereas the light color shows the area of diffusion. **(C–F)** Distribution of retrogradely labeled neurons (green) in LEC at a dorsoventral (DV) level of –7.5 mm **(C)**, and in MEC at a DV level of –5.4 mm **(E)** in horizontal sections (case: HIP2). High magnification images of the superficial layers in LEC and MEC are shown in panels **(D,F)** respectively. White arrows indicate neurons that were double-labeled with FG and CB immunoreactivity. **(G,H)** The percentage of double-labeled neurons among retrogradely-labeled neurons **(G)**, and the percentage of double-labeled neurons among the CB+ neurons **(H)** are compared between LEC and MEC (mean ± standard errors, *N* = 7; ^∗^*p* < 0.05; Wilcoxon signed rank test). Each colored dot corresponds to the value for the sample shown in panel **B**. Scale bars are 500 μm for panels **A,C,E**, and 100 μm for panels **D,F**. Sub, subiculum; DG, dentate gyrus; PaS, parasubiculum.

We first examined the distribution of CB+ neurons together with the RE+ neurons in layer II of the EC in both rats and mice. In line with previous studies, the overall distribution of CB+ and RE+ neurons differed between MEC and LEC in both species: the two types appear to be grouped in patches in MEC, while they are more or less confined to two sublayers, RE+ cells superficially (IIa) and CB+ cells deeper (IIb) in LEC (Figure 1A and Supplementary Figure 2, Tuñón et al., 1992; Fujimaru and Kosaka, 1996; Wouterlood, 2002; Ramos-Moreno et al., 2006; Varga et al., 2010; Kitamura et al., 2014; Ray et al., 2014; Leitner et al., 2016; Witter et al., 2017).

In the rat MEC, RE+ neurons were intermingled with CB+ neurons in layer II (Figure 1A and Supplementary Figures 2A,B). The reported clustering of CB+ neurons (Ray et al., 2014) was particularly striking in the dorsal MEC but not in the ventral MEC. In LEC, RE+ neurons were located almost exclusively in layer IIa, whereas CB+ neurons tended to occupy almost exclusively layer IIb. We further noticed that in LEC, RE+ neurons were often organized in patches that were separated by bundles of apical dendrites arising from CB+ neurons (Figure 1A and Supplementary Figures 2C,D).

The distribution of RE+ and CB+ neurons was different in layer II of the mouse dorsal MEC compared to that of the rat (Supplementary Figures 2A’,B’). In this layer, RE+ neurons were located in the middle and deep portions. Moreover, they were located deeper in layer II compared to CB+ neurons, which were in turn distributed in clusters in the most superficial part of this layer. At more ventral levels of MEC and in LEC this species difference was absent (Supplementary Figures 2C’,D’, Naumann et al., 2016).

### Hippocampal Projections

We first set out to analyze the projections to the hippocampus in order to confirm the previously reported projection of layer II CB+ neurons to stratum lacunosum of CA1 (Kitamura et al., 2014). We focused on the dorsal hippocampus and injected retrograde tracers in the different subfields in various combinations (*n* = 7; Figure 1B, Supplementary Figure 3). Confirming previous results, injections that include the dentate gyrus and CA1, consistently labeled many neurons in layer II and III of both LEC and MEC (Figures 1C,E), whereas injections confined to the dentate gyrus and/or CA3 result in labeling largely restricted to layer II cells (*n* = 3; data not shown). In line with previous studies, some labeled neurons were also observed in the deep layers (Cappaert et al., 2015). In LEC, the majority of the retrogradely labeled neurons were observed in layer IIa and III, with only a few in layer IIb in all cases (Figures 1C,D). In MEC, retrograde neuronal labeling was apparent throughout the depth of layers II and III. The percentage of retrogradely labeled neurons that showed CB+ co-labeling varied considerably (between 5.4 and 68.9%; Figure 1G). This large variation results from the difference of injection sites in the hippocampus. Samples which received an injection mainly in CA1 (HIP5–7) show higher percentages since retrogradely labeled neurons are preferentially located in layer III, whereas samples with an injection involving both CA1 and dentate gyrus show low percentage due to the strongly increased retrograde labeling of RE+ cells (HIP1–4). Irrespective of this substantial variation, the percentages of retrogradely labeled cells that co-labeled for CB+ were consistently lower in LEC than in MEC, 15.7 versus 37.6%, (*p* < 0.05, Wilcoxon signed rank test). In contrast, the percentage of CB+ neurons that were retrogradely labeled varied less (between 3.4 and 28.4%; Figure 1H). Yet again, the percentages in case of LEC were consistently lower than in MEC, 10.3% versus 19.0% (*p* < 0.005, Wilcoxon signed rank test). The observed consistent differences between LEC and MEC were not due to the injection position along the proximodistal axis of CA1 (Witter et al., 2000), since similar trends were observed in samples which received injections either in the proximal (HIP1) or distal CA1 (HIP2, Supplementary Figure 3). We conclude that EC projections to the hippocampus originate predominantly from neurons in layers II and III, in line with previous reports (Steward and Scoville, 1976; Witter et al., 1989a,b), with a moderate contribution of CB+ neurons in MEC, and a small contribution of CB+ neurons in LEC. These findings are thus in line with specific viral anterograde tracing data in transgenic mice that CB+ neurons in MEC and LEC project specifically to stratum lacunosum of CA1 (Supplementary Figure 4; Kitamura et al., 2014).

### Entorhinal Projections

**FIGURE 2:**
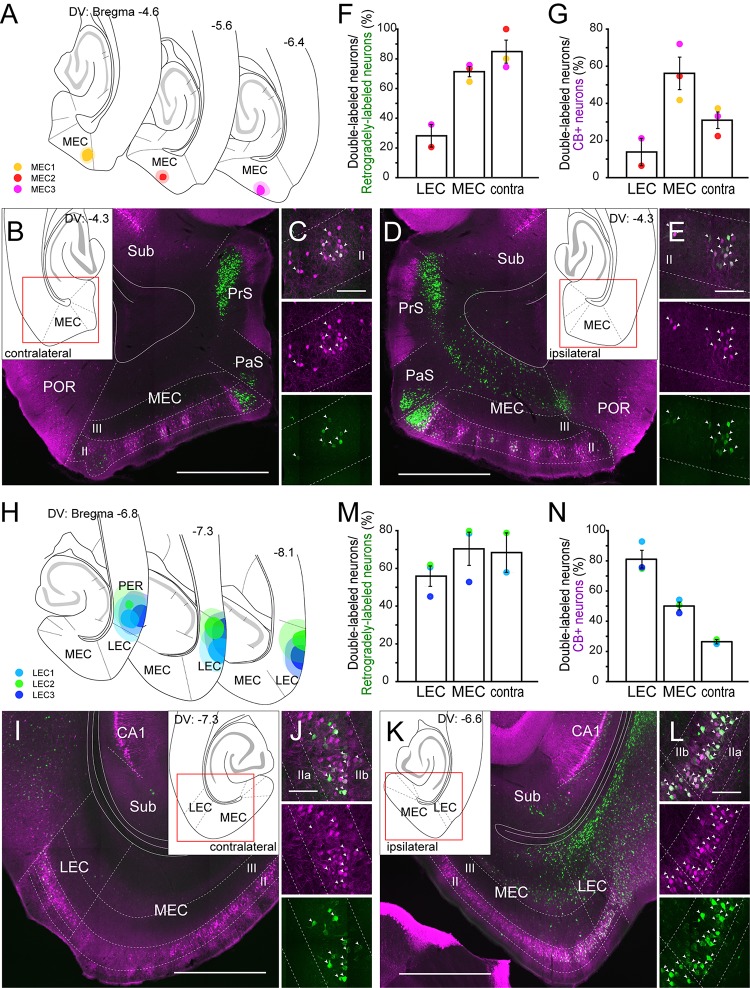
Projection of CB+ neurons to MEC **(A–E)** and LEC **(H–L)**. **(A,H)** The injection sites of retrograde tracer in samples with MEC **(A)** and LEC **(H)** injection. Each injection is illustrated with a different color. For each injection, the dark color shows the injection site while the light color shows the area of diffusion. **(B–E, I–L)** Distribution of retrogradely labeled neurons in contralateral EC **(B,I)**, and in ipsilateral EC **(D,K)** in horizontal sections (case: MEC3 for **B–E**, LEC2 for **I–L**). High magnification images of the superficial layers in contra- and ipsi-lateral EC are shown in panels **(C,J)** and **(E,L)** respectively. White arrows indicate neurons that were double-labeled with FG/FB and CB immunoreactivity. **(F,G,M,N)** The percentage of double-labeled neurons among retrogradely-labeled neurons (mean ± standard errors, **F,M**), and the percentage of double-labeled neurons among the CB+ neurons (mean ± standard errors, **G,N**) are compared between ipsilateral LEC, ipsilatarl MEC, and the contralateral counterpart. Each colored dot corresponds to the value for the sample shown in panels **(A,H)**. Scale bars are 1000 μm for panels **(B,D,I,K)**, and 100 μm for panels **(C,E,J,L)**.

To confirm the claim that CB+ neurons in MEC and LEC are a specific source of crossed projections to the contralateral EC projections (Varga et al., 2010), we analyzed the distribution of labeled neurons following injections either in the MEC (*n* = 3; Figures 2A–G) or LEC (*n* = 3; Figures 2H–N). For MEC, we injected a small volume of retrograde tracer (FB) into layer I and II (Figure 2A), since MEC CB+ neurons are known to project their axons to layer I and II of the contralateral MEC (Fuchs et al., 2016). In all three samples, many labeled neurons were observed in layer II of the contralateral MEC, and a high percentage of the contralateral labeled cells were CB+ positive (Figures 2B,C,F). Note that in addition to the labeled CB+ neurons in layer II, a substantial number of commissurally projecting neurons are found in layer III, especially in dorsal sections close to the level of the injection site (Steward and Scoville, 1976; Wouterlood, 2002; Ray et al., 2014) (data not shown). In contrast to these samples, retrograde labeling of contralateral CB+ neurons was hardly observed when the injection was placed in the deep MEC (data not shown). In addition to retrograde neuronal labeling in the contralateral EC, we observed a high percentage of double-labeling in the ipsilateral MEC (Figures 2D,E). The percentage of double-labeled neurons among the CB+ neurons was substantially lower than that of double-labeled/retrogradely-labeled neurons, and it was higher in the ipsilateral than in the contralateral MEC (Figure 2G; 56.2 versus 31.0%). In two out of three samples, retrogradely labeled neurons were also observed in the superficial layers of the ipsilateral LEC but the percentage of double-labeled neurons was lower than that seen in ipsi- and contralateral MEC.

**FIGURE 3:**
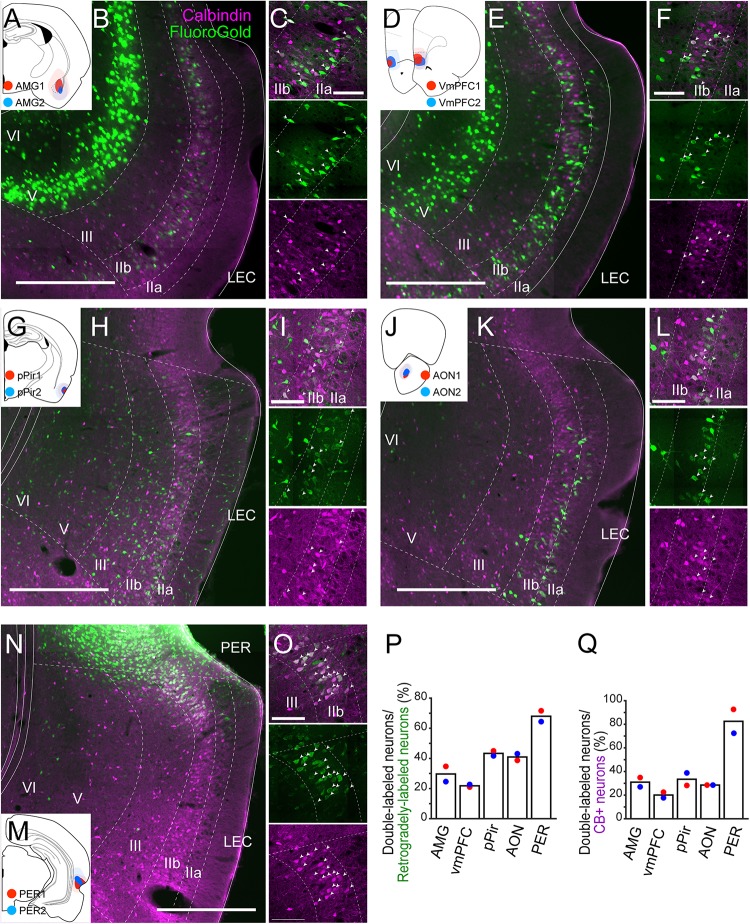
Projections of LEC CB+ neurons to telencephalic structures. FG was injected either in AMG **(A)**, vmPFC **(D)**, pPir **(G)**, AON **(J)**, or PER **(M)**. Each injection is illustrated with a different color. For each injection, the dark color shows the injection site while the light color shows the area of diffusion. Distribution of retrogradely labeled neurons in LEC after FG injection in AMG (**B,C**, case: AMG1), vmPFC (**E,F**, case: vmPFC2), pPir (**H,I**, case: pPir2), AON (**K,L**, case: AON2), and PER (**N,O**, case: PER2) in coronal sections. White arrows indicate neurons that were double-labeled with FG and CB immunoreactivity. **(P,Q)** The percentage of double-labeled neurons among retrogradely-labeled neurons (*N* = 2 each, **P**), and the percentage of double-labeled neurons among the CB+ neurons (*N* = 2 each, **Q**) in LEC. Scale bars are 500 μm for panels **(B,E,H,K,N)**, and 100 μm for panels **(C,F,I,L,O)**.

In case of LEC, we injected the retrograde tracer (FG) into the superficial layers of LEC (Figure 2H). In two out of three samples, many labeled neurons were observed in the contralateral EC, and a high percentage of the contralateral layer II cells were CB+ (Figures 2I,J,M). A high percentage of retrogradely labeled neurons were also double-labeled in ipsilateral LEC and MEC (Figures 2K,L,M). Similar to the case of MEC injection, the percentage of double-labeled neurons among the CB+ neurons was higher in the ipsilateral than in the contralateral LEC (Figure 2N; 81.1 versus 26.5%). The labeling originating from the ipsilateral interconnections between the LEC and MEC, however, was different between the MEC- and LEC-injection cases. For the projections of MEC CB+ neurons to LEC we noted a higher percentage of double-labeled neurons than the other way around (Figures 2F,G,M,N; 70.4 versus 28.2% for double-labeled/retrogradely-labeled neurons, 50.1 versus 13.8% for double-labeled/CB+ neurons). Note that all three injections aimed to target LEC leaked into the perirhinal cortex (PER), implying that labeled neurons in the contralateral LEC and ipsilateral EC could be due to this unintended leakage. However, retrograde injections confined to PER did not results in labeled neurons in contralateral LEC and ipsilateral MEC (Figures 3M–O, Supplementary Figure 6L), so we find it likely that the labeling in these areas is due to injecting in LEC. In contrast, the ipsilateral LEC retrograde labeling might be confounded by neurons that are retrogradely labeled due to PER involvement (see also below other telencephalic projections).

We also analyzed the distribution of labeled neurons following injections in the border region between LEC and MEC (*n* = 8; Supplementary Figure 5A). Relatively high percentages of the retrogradely labeled neurons were CB+ in ipsi- and contralateral of LEC and MEC, although the percentage was significantly higher in ipsilateral MEC than in LEC (Supplementary Figures 5B–F; 47.5 versus 31.3%; Friedman test *p* = 0.0148; Dunn’s multiple comparisons post-test, *p* < 0.05). In contrast, similar to the results shown in Figure 2, the reverse percentage (percentage of the double-labeled neurons among the CB+ neurons) was significantly higher in ipsilateral than in contralateral EC (Supplementary Figures 5B–E,G; Friedman test *p* = 0.0002; Dunn’s multiple comparisons post-test, *p* < 0.05 for ipsi-LEC vs. contra-LEC, *p* < 0.01 for ipsi-MEC vs. contra-MEC). In one case, we injected FG in the ipsilateral EC and CTB-555 into the contralateral EC (EC5), resulting in some double labeled CB+ neurons, indicating that some CB+ neurons have projection to both ipsi- and contralateral EC (Supplementary Figure 5H).

### Other Telencephalic Projections

Neurons in the EC in rodents project to a number of telencephalic domains, other than the EC and hippocampus. These include projections to olfactory domains, multimodal cortical areas as well as subcortical areas (Swanson and Köhler, 1986; Insausti et al., 1997; Kerr et al., 2007; Cappaert et al., 2015). Although many of these entorhinal projections originate from neurons in layer Va (Insausti et al., 1997; Sürmeli et al., 2015; Ohara et al., 2018), contributions from superficial layers II and III have also been reported, in particular in case of projections to olfactory and medial prefrontal areas and the amygdaloid complex (Shipley and Adamek, 1984; Insausti et al., 1997; Cappaert et al., 2015). In recent studies in mice, projections from CB+ layer II neurons in LEC to olfactory cortex and olfactory bulb have been described (Leitner et al., 2016).

To examine the contribution of the EC CB+ layer II neurons to these potential telencephalic projections, retrograde tracers were injected into telencephalic targets of EC, and the distribution of the retrogradely labeled neurons was examined in LEC and MEC. We placed injections in prelimbic cortex (PrL; *n* = 2), anterior piriform cortex (APir; *n* = 2), ventral orbitofrontal cortex (OFC; *n* = 3), nucleus accumbens (NAc; *n* = 2), anterior insular cortex (AIC; *n* = 2), retrosplenial cortex (RSC; *n* = 3), postrhinal cortex (POR; *n* = 2), ventral medial prefrontal cortex (vmPFC; *n* = 2), amygdaloid complex (AMG; *n* = 2), anterior olfactory nucleus (AON; *n* = 2), posterior piriform cortex (pPir; *n* = 2), and PER (*n* = 2; Supplementary Figure 6). In all cases, retrogradely labeled neurons were present mainly in layer Va of EC. In a number of cases, we observed additional retrogradely labeled neurons in LEC layer IIb. These cases had injections in vmPFC including infralimbic and medial orbitofrontal and dorsal peduncular cortex (IL/DP/MO), AMG, AON, pPir, and PER (Supplementary Figures 6H–L). No superficially located MEC neurons were labeled following injections in any of these five areas. Therefore, we further examined the co-localization of retrograde-labeling and CB+ labeling only in LEC (Figure 3). In samples with an injection in AMG (*n* = 2; Figures 3A–C), vmPFC (*n* = 2; Figures 3D–F), pPir (*n* = 2; Figures 3G–I), and AON (*n* = 2; Figures 3J–L), the percentages of CB+ neurons among the retrogradely labeled LEC neurons were 29.7, 21.9, 43.4, and 40.9%, respectively (Figure 3P). The percentages of CB+ neurons that were retrogradely labeled were 31.0, 20.1, 33.5, and 28.5%, respectively (Figure 3Q). Massive retrograde labeling was also observed in LEC layer IIb after FG injection in PER (*n* = 2; Figures 3M–O). Although the percentage of double-labeled neurons was high in this case (Figures 3P,Q), the distribution of retrogradely labeled neurons was restricted to the very dorsal portion of LEC close to the border of PER (Figure 3N).

We subsequently assessed whether there are LEC CB+ neurons that send collateralized projections to two targets as previously reported in case of olfactory and contralateral projections (Leitner et al., 2016). Injections of two different fluorescent chemical tracers in vmPFC and ipsilateral EC resulted in a low number of double labeled neurons (Supplementary Figure 7). Such collateralization of the local projecting LEC superficial neurons was further examined by an AAV double infection approach. In this approach, retrograde-infecting AAV, expressing Cre recombinase (AAV6-Cre) was injected into the rostral LEC and a Cre-dependent reporter AAV that expresses EYFP after recombination (AAV1/2-EF1α-DIO-EYFP) was injected into the border of LEC and MEC (*n* = 2, Supplementary Figures 8A,B,I). EYFP-expressing somata were distributed within the superficial layer of LEC, mainly in layer IIb, and approximately 40% of them were CB+ (Supplementary Figures 8C,J). In addition to a massively labeled fiber plexus in ipsilateral LEC (LI–III) and MEC (LI), labeled fibers were observed in olfactory areas, including AON and pPir (LI), PER (LI), and vmPFC especially in dorsal peduncular cortex (LI–III, Supplementary Figures 8D–H, K–P). Massive labeling of passing fibers as well as terminal-like labeling was also observed in the endopiriform nucleus and AMG. Since our retrograde tracing experiments show that such extrinsic projections mainly originate from layer IIb and not from layer IIa/III, we conclude that local projecting LEC CB+ neurons also send collaterals to extrinsic regions. The data also indicate the endopiriform nucleus as a possible target of LEC CB+ neurons. We also tested whether CB+ neurons might contribute to projections to the medial septum, in view of a recent mouse study, in which it was reported that MEC CB+ neurons project to MS (Fuchs et al., 2016). In our rat study, injections in the septal complex did produce labeling in LII of EC but the retrogradely labeled neurons were sparsely observed only in ventral EC (Supplementary Figures 9A–F, Alonso and Köhler, 1984), and the colocalization with CB+ was also sparse. Finally, we placed retrograde tracer injections into the thalamic nucleus reuniens (*n* = 3), but these did not result in labeled neurons in either LEC or MEC (Supplementary Figures 9G,H). This is in line with a previous study showing that rostromedial or caudomedial reuniens hardly receive input from EC and that the few EC neurons projecting to the rostrolateral reuniens are mainly located in deep layers (McKenna and Vertes, 2004).

The CB+ population in EC comprise GABAergic neurons in addition to glutamatergic excitatory neurons (Wouterlood and Jasperse, 2001), and therefore, CB+ inhibitory neurons may contribute to the extrinsic and intrinsic projection shown above. To investigate this possibility, we injected retrograde tracers into the dorsal CA1 and contralateral MEC of GAD67 transgenic mouse line expressing GFP (Tanaka et al., 2003), and examined the distribution of retrogradely labeled neurons in EC layer II (*n* = 2, Supplementary Figure 10). Similar to the results observed in rats (Figures 1,2), CB+ neurons of both MEC and LEC were retrogradely labeled by the tracer injected into the dorsal CA1 and contralateral EC (Supplementary Figures 10C–F). CB+ entorhinal neurons, ipsilateral to the Fast Blue injection in MEC, were also retrogradely labeled (Supplementary Figures 10G–I). We further observed CB+ inhibitory neurons which are double-positive for CB and GAD67-GFP. These neurons tend to have high levels of CB immuno-labeling. We did not find any CB+ inhibitory neurons that were retrogradely labeled in contralateral MEC (Supplementary Figure 10D), contralateral LEC (Supplementary Figure 10F), and in ipsilateral LEC (Supplementary Figure 10H). We did find a very low presence of triple-labeled neurons only in MEC ipsilateral to the fast blue injection (Supplementary Figure 10I). Together, these results indicate that CB+ inhibitory neurons contribute to the intrinsic projections, but not to the long-range extrinsic projections. In other words, the long-range extrinsic projections of CB+ entorhinal neurons likely originate solely of the excitatory set of CB+ neurons.

**FIGURE 4:**
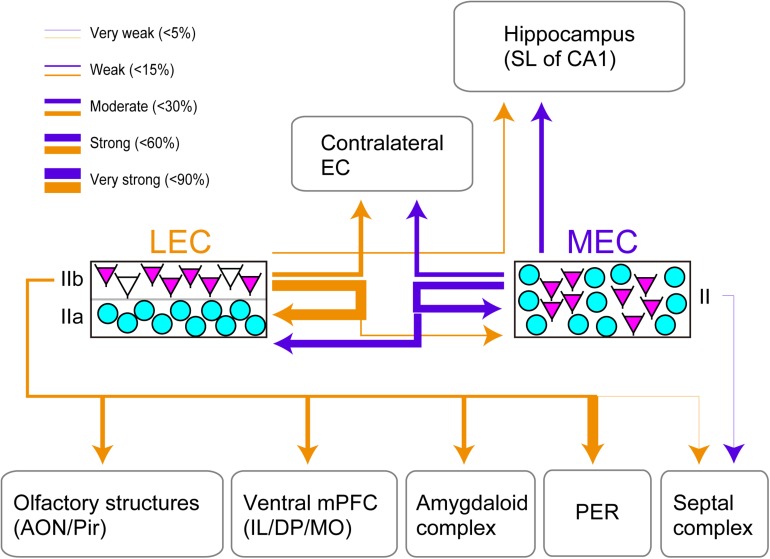
Summary of the projection of CB+ neurons in LEC and MEC. EC Layer II neurons which comprise CB+ neurons (magenta) and RE+ neurons (cyan) are shown. The arrows indicate the projections originating from the LEC CB+ neurons (yellow) and the MEC CB+ neurons (purple). Note that the arrows do not show the laminar targets of the ipsi- and contralateral EC projections. The thickness of the lines is based on the percentage of double-labeled neurons/CB+ neurons. Note that although the projection from LEC CB+ cells to PER seems strong, this projection originates only from a restricted very dorsal portion of LEC.

This study provides the first systematic and quantitative analysis of efferent projections of CB+ neurons in layer II of both LEC and MEC (Figure 4). We conclude that CB+ neurons in both LEC and MEC are the source of widespread cortical and subcortical projections, partially mirroring the known projections of EC as well as the well-established differences between efferent projections of LEC and MEC. The majority of MEC layer II CB+ neurons are intrinsic projecting neurons targeting LEC (50.1% of the total population), MEC (56.2%), and contralateral MEC (31.0%), and the remainder contribute to hippocampal projections (19.0%). In LEC, these percentages are 81.1% to LEC, 13.8% to MEC, 26.5% to contralateral LEC, and 10.3% to hippocampus, additionally contributing substantially to projections to AMG (31.0% of the CB+ neurons), vmPFC (20.1% of the CB+ population), olfactory structures (28.5% to AON and 33.5% to pPir), and PER (82.5% of the CB+ population). We further report that EC efferents, originating exclusively from layer V neurons, are less commonly associated with a parallel CB+ layer II pathway. Most strikingly, our data point to CB+ neurons as key elements of local ipsi- and contralateral EC circuitry.

## Discussion

The connectivity and functional relevance of CB+ neurons became an issue of importance ever since it was described that in MEC layer II there is a substantial number of those neurons. The first report described that CB+ neurons are in majority excitatory pyramidal neurons contributing to extrinsic efferent projections of MEC to contralateral MEC by showing that 88.9% of the retrogradely labeled neurons were CB+ positive (Varga et al., 2010). This study, however, did not describe the proportion of the CB+ neurons contributing to this projection. This was also the case in the papers which later reported the projections from MEC CB+ neurons to the medial septum (Fuchs et al., 2016) and the projections of LEC CB+ neurons (Leitner et al., 2016). Further projections of MEC CB+ neurons to CA1 were revealed by using a transgenic mouse line (Kitamura et al., 2014) or to ipsilateral MEC (Zutshi et al., 2018). These studies also did not show the proportion of the CB+ neurons contributing to each of these projections. All these studies therefore lead to two potential misinterpretations: (i) that a very substantial percentage of CB+ neurons project to these targets and (ii) that the majority of CB+ neurons therefore project to all of these targets. Our results show that this is not the case. First of all, the large majority of crossed and commissural projections originate from CB- neurons in layer III of the EC, which is in line with previous reports (Steward and Scoville, 1976; Köhler et al., 1978). More significant is the fact that the CB+ neurons which project to the contralateral EC, the hippocampus, and the medial septum are only part of the total CB+ population (26.7, 10.3, and 4.2% respectively for LEC CB+ neurons, and 31.0, 19.0, and 2.1% respectively for MEC CB+ neurons). We further show that the LEC CB+ neurons, contribute to a number of additional long-range projections not reported previously. Essentially all of the long-range extrinsic projections originate from excitatory CB+ neurons. A substantial part of the CB+ population projects intrinsically (50.1% for LEC CB+ neurons, and 56.2% for MEC CB+ neurons). This intrinsic projection mainly originates from CB+ excitatory neurons though CB+ inhibitory neurons do contribute to this local innervation.

The present semiquantitative findings can only be revealed by the extensive retrograde tracing approach as applied in this study, and not by the use of CB-specific transgenic mice. Anterograde tracing experiments using transgenic mouse lines are indeed powerful to examine the detailed connectivity of specific cell types with homogeneous projections. However, this approach is not ideal when it comes to CB+ neurons which, as we show here, are a heterogenous population with diverse projections, since such an approach would simply label all projections originating in CB+ neurons. Of course, retrograde tracing approaches also have limitations, and the number of retrogradely labeled neurons vary regarding to the amount of the tracer injected, the size of the injection site, as well as the sensitivity of tracer detection. The retrograde tracer can be taken up not only by the axon terminal but also by passing fibers, which can result in false positive labeling (Schmued and Fallon, 1986). The estimates of the connectivity shown in this study are thus semi-quantitative, but still unequivocally show the unique projection system originating from CB+ neurons in both LEC and MEC.

We further show that CB+ neurons in both EC subdivisions contribute sparsely to a variety of projections outside EC, most, if not all of which have a shared origin in layer III and in some instances also in layer V. These include projections to mPFC, olfactory telencephalic structures, the amygdaloid complex, endopiriform nucleus and septal complex (Cappaert et al., 2015). Projections, known to originate almost exclusively from layer V, including those targeting the cingulate and retrosplenial cortex, the orbitofrontal and insular cortex, as well as the nucleus accumbens (Cappaert et al., 2015), do not seem to have much of an accompanying CB+ pathway. All these projections, irrespective of whether they originate in MEC or LEC, have their origin in a single layer of genetically defined neurons, generally referred to as layer Va (Sürmeli et al., 2015; Ohara et al., 2018). We recently reported that layer Va neurons contribute little to local connections, in contrast to layer Vb neurons (Ohara et al., 2018). Aside from showing that the LII CB+ excitatory population comprises neurons with diverse projections, the three main messages in this paper are: (i) the largest percentage of layer II CB+ neurons contribute to intrinsic local projections, thus representing a yet not described group of excitatory interneurons, (ii) the CB+ neurons, including the local projecting population, tend to collateralize, targeting multiple targets, and (iii) CB+ neurons in LEC and MEC show strikingly different overall projection patterns, largely replicating the overall differences of efferent projections between the two entorhinal subdivisions.

### CB+ Neurons Are Key Neuronal Intrinsic Network Elements

The intrinsic projections of CB+ neurons distribute bilaterally, within the area of origin and its commissural counterpart, but also contribute to unilateral interconnections between LEC and MEC. Although this is true in case of CB+ neurons in both LEC and MEC, there are striking differences in the numerical weight of these intrinsic projections (see below). This striking widespread intrinsic connectivity and sparse hippocampal CA1 connectivity makes layer II CB+ neurons very different from their counterparts, the RE+ neurons. The latter have strong and widespread projections to the dentate gyrus, CA2 and CA3 and based on *in vitro* studies, their local connectivity apparently is rather restricted to a small domain around the cell body (Pastoll et al., 2012; Couey et al., 2013; Schmidt et al., 2017; Nilssen et al., 2018). This notion is supported by the present retrograde data. Moreover, unlike CB+ neurons, the RE+ population does not seem to contribute substantially to any of the other extrinsic EC projection targets. It is of interest to note that in our hands, injections of retrograde tracers in EC, vmPFC and AMG resulted in many retrogradely labeled LIIb neurons, which seemed negative for CB+. Although we cannot exclude that this is an artifact of our immunohistochemical procedures, we suggest that these observations indicate that there might yet be another neuron type in LEC LII (reelin-negative/CB-negative pyramidal cell) of which the identity needs to be determined. A potential candidate might be the much sparser population of calretinin-positive pyramidal neurons, known to be present in layer IIb (Wouterlood et al., 2000), but we lack conclusive data on these neurons. It is presently unknown how the morphologically described cell types in layer II of both LEC and MEC (Canto and Witter, 2012a, b; Cappaert et al., 2015; Fuchs et al., 2016; Leitner et al., 2016) relates to the class of calretinin + neurons.

It is also well established that CB+ neurons have local connections different from RE+ neurons. Whereas RE+ neurons preferentially reciprocally connect with PV + interneurons, CB+ are connected to interneurons expressing the 5HT3a receptor in case of MEC, and these likely represent CCK expressing basket cells (Varga et al., 2010; Burgalossi and Brecht, 2014; Fuchs et al., 2016). Moreover, MEC CB+ neurons reportedly receive specific inputs from cholinergic neurons in the medial septal complex and also from parasubicular neurons that apparently avoid RE+ neurons (Ray et al., 2014; Tang et al., 2016). It also has been reported that the MEC microcircuitry differs between the two cell types, such that layer II stellate cells receive more superficial input than layer II pyramidal cells, and pyramidal cells receive more deep layer input than stellate cells (Beed et al., 2010). Both cell types share however a dominant distribution of their axons to layer I and superficial layer II, be it that the range of these projections is very different, as mentioned above. The present data on the preferred termination of CB+ local intrinsic ipsilateral projections in layer I is in line with previous reports (Köhler, 1986, 1988; Ray et al., 2014; Fuchs et al., 2016; Leitner et al., 2016), and holds true for the contralateral projections in case of MEC as well (present data; Blackstad, 1956; Zheng et al., 2014; Fuchs et al., 2016). Our data further show that the long-range projections from CB+ neurons in LEC targeting MEC, show a similar laminar distribution. Own unpublished results indicate that the opposite projection from MEC to LEC shows a terminal preference for layer II (Doan et al., 2016). Finally, CB+ pyramidals are known to provide excitatory input to the RE+ stellate cells both directly (Winterer et al., 2017) and indirectly through the CB+ intermediate pyramidal cells (Fuchs et al., 2016). A comparable wiring scheme is likely applicable to LEC (Witter et al., 2017). Since CB+ neurons also provide feed-forward inhibition to the CA1 pyramidal cells (Kitamura et al., 2014), we propose that activity in the CB+ population might switch the information flow in the EC-hippocampal system from the EC layer III-CA1/subiculum direct pathway to the EC layer II-DG/CA3 indirect pathway.

Intrinsic connectivity in EC of the rat not only originates from layer II CB+ neurons but also from neurons in layers III –VI and distributes in layers I–V (Köhler, 1986, 1988; Dolorfo and Amaral, 1998). This seems to hold true in other species as well (Witter et al., 1986, 1989b; Chrobak and Amaral, 2007). Our data for both LEC and MEC, in line with previous reports (Köhler, 1986, 1988; Fuchs et al., 2016; Leitner et al., 2016), thus indicate an interesting differentiation between the two systems. Whereas layer II CB+ neurons originate projections that preferentially terminate in layer I and superficial layer II, the projections to the deeper layer seem to originate mainly from neurons in layers III and V.

### CB+ Projections Collateralize

The percentages of CB+ neurons that project to the identified projection targets for both LEC and MEC add up to way over 100%. In case of MEC we identified over 150% of the population of CB+ neurons and in LEC we identified over 300% based on single tracing experiments. We take these numbers as an indication that CB+ neurons in EC give rise to strongly collateralized projections and corroborated that contention by showing that retrograde double labeling occurs in case of injections in two targets. This is in line with previous reports. Morphologically, the populations of CB+ neurons in both LEC and MEC comprise two different neuronal types, pyramidal neurons and oblique/intermediate pyramidals (Fuchs et al., 2016; Leitner et al., 2016). It might thus be the case that the two morphologically different CB+ neurons can be equated with two populations of projection-selective neurons, for example one bilaterally intrinsic and one extrinsic. This might seem a likely scenario since in MEC, CB+ projection neurons projecting to contralateral MEC and the medial septum are colocalized in the same cluster, but single cells do not seem to collateralize to both targets (Fuchs et al., 2016). Supporting but yet insufficient data have been obtained in LEC, where single CB+ neurons have been shown to project to the piriform cortex and the olfactory bulb, but no evidence was presented that these also project intrinsically, neither ipsi- nor contralaterally (Leitner et al., 2016), and our results showing that CB+ neurons collateralize to target both ipsilateral and contralateral LEC. Conflicting with this notion are our present observations that single CB+ neurons can project to vmPFC and ipsilateral LEC. It is therefore not possible to relate the two morphologically defined CB+ neurons to their projection patterns. Our data further indicate that the level of collateralization in LEC is higher than in MEC, likely reflecting the increased number of CB+ projecting targets in case of LEC.

### CB+ Projections From LEC Are More Diverse Than the Ones From MEC

We report striking differences between LEC and MEC in that CB+ MEC projections mainly reach CA1 and bilaterally target MEC, as well as contributing substantially to projections to LEC. In contrast, CB+ projections from LEC to CA1 are less pronounced than their MEC counterpart, whereas commissural projections are comparable. Projections of LEC CB+ neurons to MEC are numerically much weaker than the other way around (13.8 versus 50.1%). Further, LEC CB+ neurons contribute substantially to projections to targets not reached by MEC CB+ neurons. These targets include the amygdala, the medial prefrontal cortex and the perirhinal cortex. In other words, MEC is more parahippocampal/hippocampal centric, whereas LEC prefers other telencephalic structures over parahippocampal and hippocampal projections. This is in line with the overall excitatory connectivity patterns of LEC and MEC (Witter et al., 2017; Nilssen et al., 2019). Interestingly a similar difference in connectivity patterns have been reported with respect to inputs to interneuron populations in MEC versus LEC as well (Jacobsen et al., 2018).

These results are of interest when combined with two additional features. First, MEC CB+ neurons receive inputs from deep layers, which likely convey information processed in the hippocampus (Beed et al., 2010). Second it has been reported, using the isolated guinea pig *ex vivo* brain preparation, that olfactory stimulation resulted in a sequential activation in LEC, hippocampus and MEC, followed by LEC (Biella and de Curtis, 2000). Since we here show that reciprocal connections of LEC and MEC are unequal in strength, in favor of the MEC to LEC ones, and that LEC CB+ neurons provide an additional preferential projection to telencephalic structures including the olfactory regions, we suggest that hippocampal information may be processed first in MEC and subsequently in LEC followed by telencephalic structures. In case this turns out to be a generalizable trait, inputs arriving in LEC will, after hippocampal processing, not be returned to LEC but will be processed hierarchically from MEC to LEC and further downstream.

## Conclusion

In conclusion, CB+ neurons in MEC and LEC are the source of a widespread intrinsic excitatory projection, connecting ipsilateral LEC and MEC to contralateral LEC and MEC respectively, as well reciprocally connecting LEC and MEC within one hemisphere. Such local circuits of MEC LII pyramidal cells are critical for the precise firing location of grid cells (Zutshi et al., 2018). In addition to this main projection, we showed that the long-range projections of CB+ neurons outside EC differ between LEC and MEC. Although such extrinsic projections are numerically-weaker than the intrinsic ones, a high-degree of cellular specificity can still be present, such as the selective targeting of interneurons in CA1 stratum lacunosum which controls temporal association memory (Kitamura et al., 2014). Although plausible, whether the intrinsic and extrinsic projections of CB+ neurons specific for the two entorhinal subdivisions contribute to the functional difference between LEC and MEC require further investigation.

## Data Availability Statement

All datasets generated for this study are included in the manuscript/Supplementary Files.

## Ethics Statement

The animal study was reviewed and approved by the Center for Laboratory Animal Research of Tohoku University and the Norwegian Animal Research Authority.

## Author Contributions

SO and MW conceived the study design. SO, MG, KI, CB, and TD collected and analyzed the experimental data. TK and KM produced AAV vectors. SO and MG carried out all the quantifications. All authors contributed to the discussions that resulted in this article, which was written by SO and MW, and approved the final version of the manuscript.

## Conflict of Interest

The authors declare that the research was conducted in the absence of any commercial or financial relationships that could be construed as a potential conflict of interest.

## Footnotes

1 http://rsb.info.nih.gov/ij
