# Supplementary material for: Entorhinal Layer II Calbindin-Expressing Neurons Originate Widespread Telencephalic and Intrinsic Projections
Source: Front Syst Neurosci. 2019 Oct 15;13:54. doi: 10.3389/fnsys.2019.00054 (PMC6803526; doi:10.3389/fnsys.2019.00054)
Supplement: Supplementary file 1 [file Data_Sheet_1.PDF]

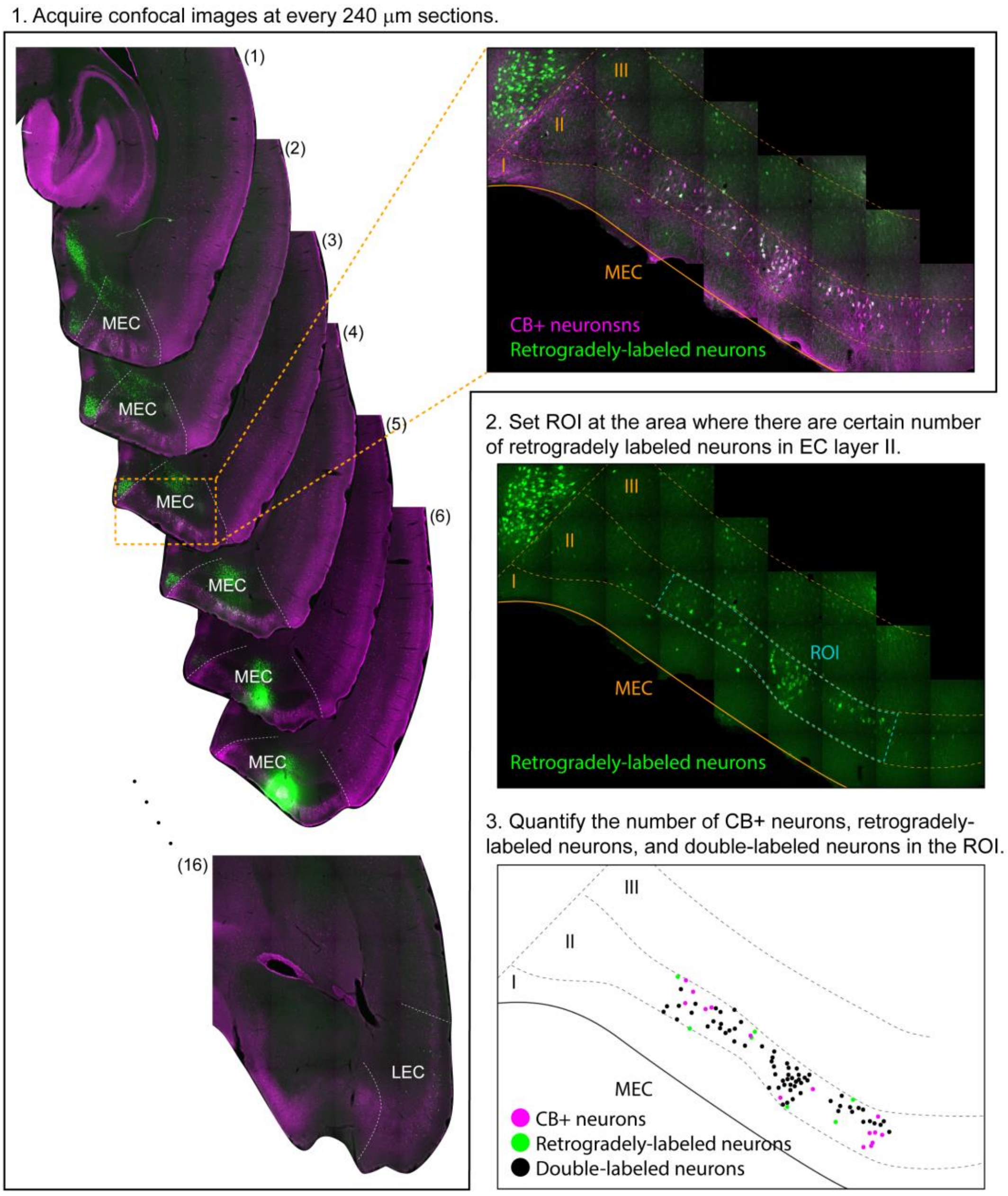

4. Calculate the percentage of “Double-labeled neurons/CB+ neurons” and “Double-labled neurons/ Retrogradely-labeled neurons”.

CB+ neurons	Retrogradely-labeled neurons	Double-labeled neurons	Double-labeled neurons/ CB+ neurons (%)	Double-labeled neurons/ Retrogradely-labeled neurons (%)
97	71	64	65.98	90.14

Supplementary Figure 1. Schematic diagram illustrating the quantitative analysis of the colocalization of CB immunolabeling and retrograde labeling. Samples were analysed in sections that were 240 μm apart, using one z-level of the confocal image (Step 1). We selected a Region of Interest (ROI) where there were sufficient retrogradely labeled neurons in EC layer II (Step 2). Subsequently, the retrogradely labeled neurons and immunohistochemically stained CB+ neurons were counted in this ROI (Step 3). The percentage of “Double-labeled neurons/CB+ neurons” and “Double-labeled neurons/Retrogradely-labeled neurons” were calculated and compared between MEC and LEC (Step 4).

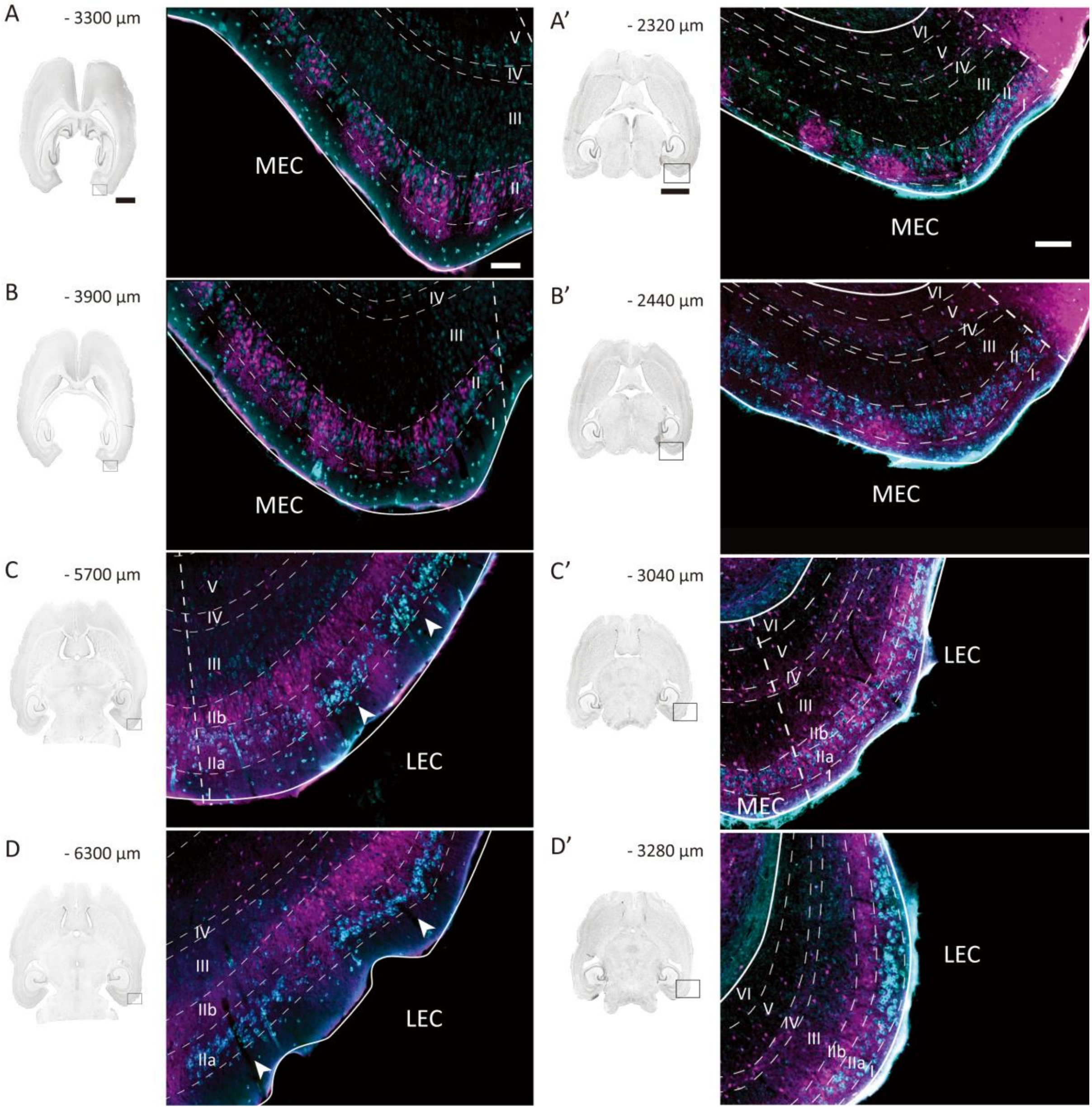

Supplementary Figure 2. Distribution of neurons immunoreactive for RE (cyan) and CB (magenta) in rat (A–D) and mouse (A'–D') EC at four different levels from dorsal to ventral. Left panel represents Nissl stained sections; right panel shows magnifications of parts of immunostained images corresponding with the boxed areas in the Nissl stained sections. Arrows in C–D: patches of reelin-positive neurons separated by bundles of dendrites arising from CB-positive neurons. Scale bars A and A' (apply to all sections in the same column): black scale bar equals 2000 µm; white scale bar equals 100 µm.

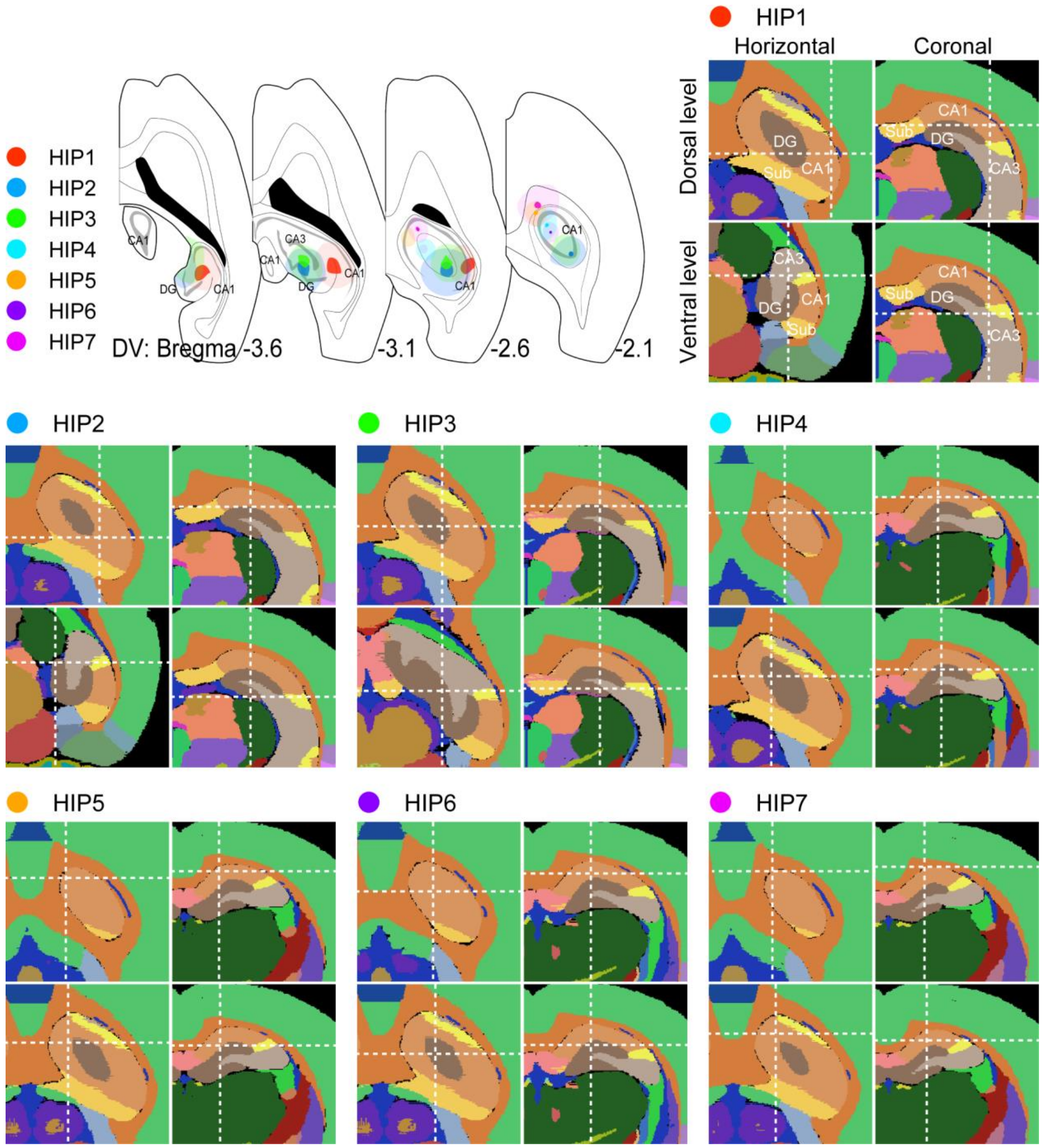

Supplementary Figure 3. The injection sites in the hippocampus were reconstructed in coronal sections using the Waxholm space based three-plane architectonic atlas of the rat hippocampal region (Boccara et al., 2015; Kjonigsen et al., 2015). Intersection of dotted lines represents the position of the injection site. Location of the injection sites are shown at the dorsal (top panel) and the ventral level (bottom panel) for each case. Left panels show the injection position in horizontal sections, whereas right panels show the corresponding location of the injection site in coronal sections.

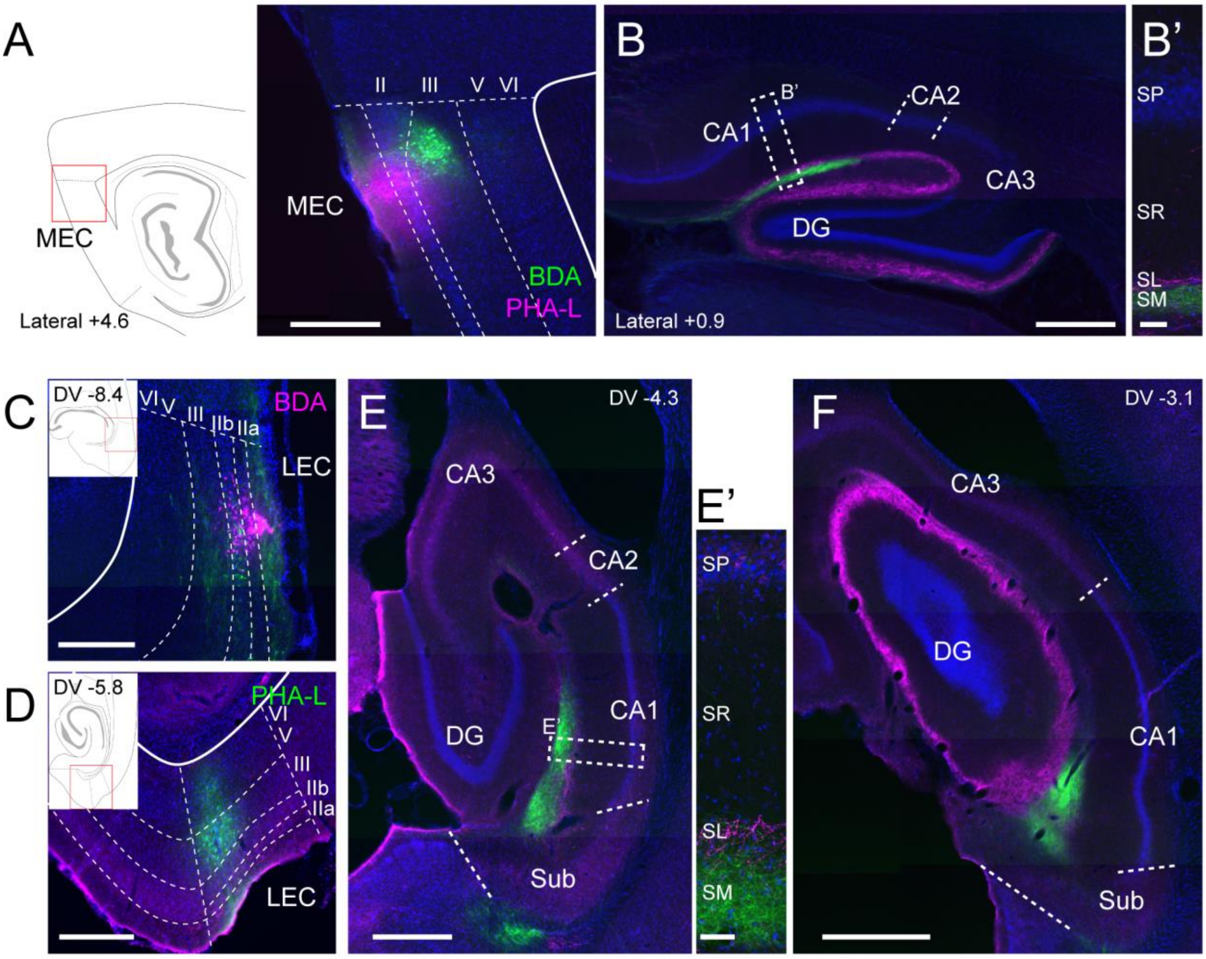

Supplementary Figure 4. Anterograde tracing from EC superficial layers to the hippocampus. (A, B) Line drawing of a sagittal sections indicating the position of the image showing the injection sites of the two anterograde tracers, PHA-L and BDA, in MEC layer II and III respectively (A). The distribution of labeled fibers (PHA-L: magenta , BDA: green) as seen in the hippocampus (B) in a sagittal section. (B') High power image of the boxed area indicated in B, showing the differential projections of layer III neurons to stratum moleculare (SM) and from layer II neurons to stratum lacunosum (SL). (C–F) Injection sites of BDA and PHA-L into LEC layer II and III respectively (inset shows a schematic line drawing of part of a horizontal section, indicating the position of the image; C, D) The distribution of labeled fibers (BDA: magenta, PHA-L: green) as seen in the hippocampus (E, F) in horizontal sections. (E') High power image of the boxed area indicated in E, showing the differential projections of layer III neurons to SM and from layer II neurons to SL. Scale bars are 1000 µm for (E) and (F), 500 µm for (A), (B), (C), and (D), and 50 µm for (B') and (E'). Abbreviations: SP, stratum pyramidale; SR, stratum radiatum.

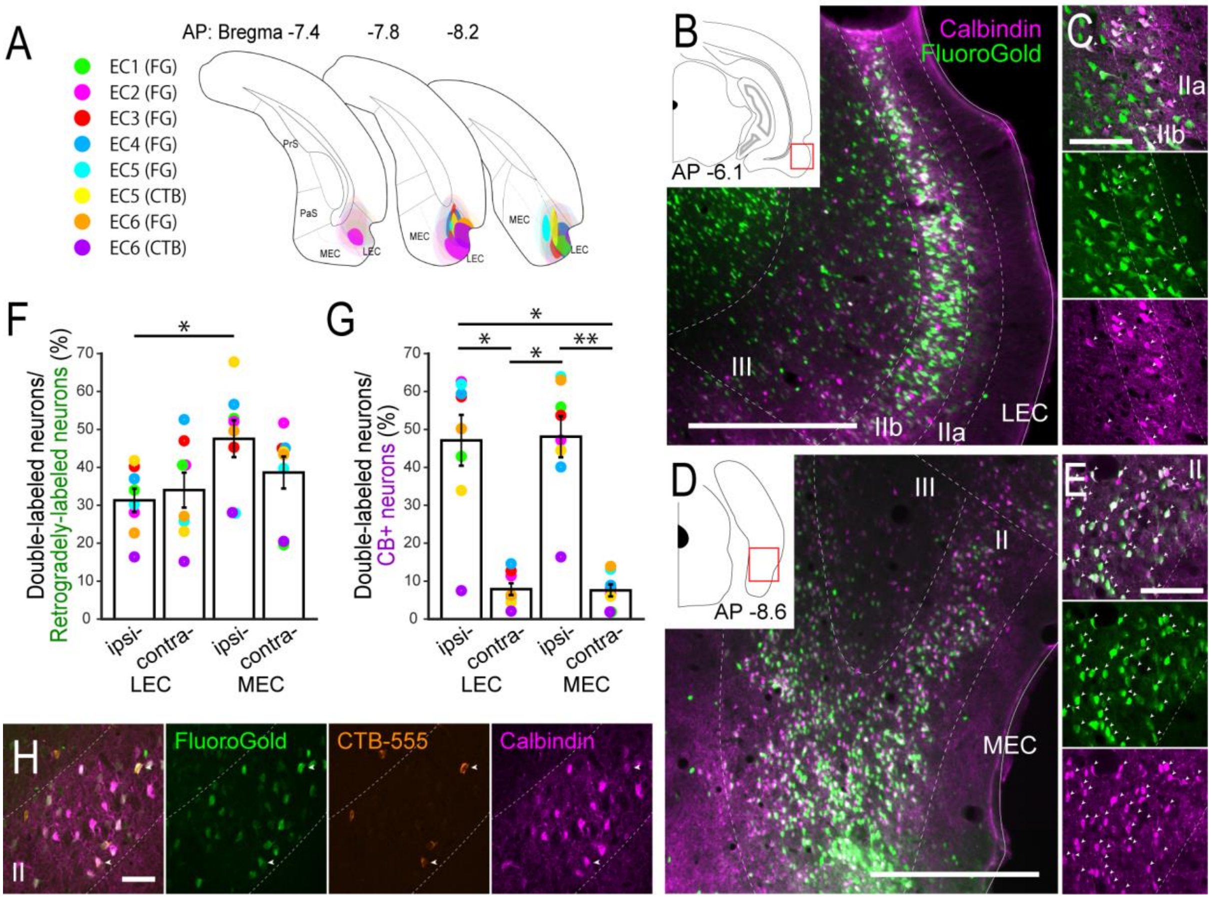

Supplementary Figure 5. Projection of CB+ neurons in LEC and MEC to the ipsi- and contralateral EC. (A) The injection sites of retrograde tracer injections in EC (n = 8 in 6 animals) are illustrated with a different color in three coronal sections taken at different anteroposterior (AP) levels from bregma. The dark color shows the injection site and the light color shows the area of tracer diffusion. CTB-555 was injected in the contralateral EC in case “EC5” and “EC6”, but the injection sites are illustrated in the same hemisphere for simplicity. (B–E) Distribution of retrogradely labeled neurons in ipsilateral LEC at an AP level of -6.1 mm (B), and in ipsilateral MEC at an AP level of -8.6 mm (D) in coronal sections (case: EC2). High magnification images of the superficial layers in LEC and MEC are shown in (C) and (E) respectively. White arrows indicate neurons that were double-labeled with FG and CB immunoreactivity. (F, G) The percentage of double-labeled neurons among the retrogradely-labeled neurons (F), and the percentage of double-labeled neurons among the CB+ neurons (G) are compared between LEC and MEC in the ipsilateral and contralateral side of the injection (mean ± standard errors, N = 8; *$p < 0.05$, **$p < 0.01$, Friedman test followed with Dunn's multiple comparison post test). Each colored dot corresponds to the value for the sample shown in (A). (H) High magnification images of MEC in case “EC5”, which received a FG injection in the ipsilateral EC and a CTB-555 injection into the contralateral EC. White arrows indicate neurons that were triple-labeled with FG, CTB-555, and CB immunoreactivity. Scale bars are 500 μm for (B) and (D), and 100 μm for (C), (E), and (H).

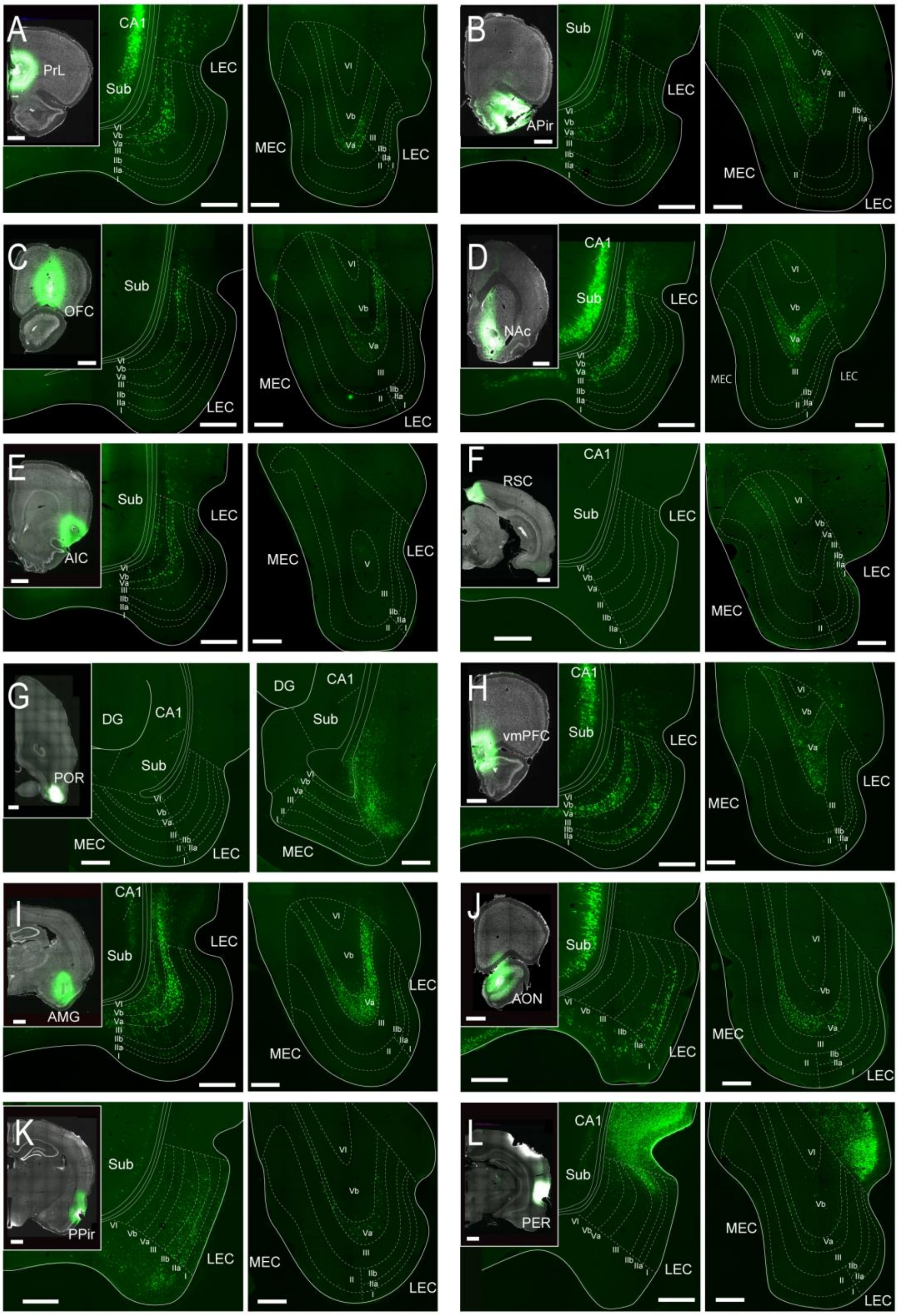

Supplementary Figure 6. Distribution of retrogradely labeled neurons in LEC and MEC after FG injection into telencephalic structures. The injection site is shown in the inset, and the distribution of retrogradely labeled neurons in the LEC and MEC are shown in the left and right panel, respectively, for each injection case. The images are from coronal sections except for (G), which shows horizontal sections. Scale bars are 1000 µm in the injection site (inset), and 500 µm in LEC and MEC images (left and right panel).

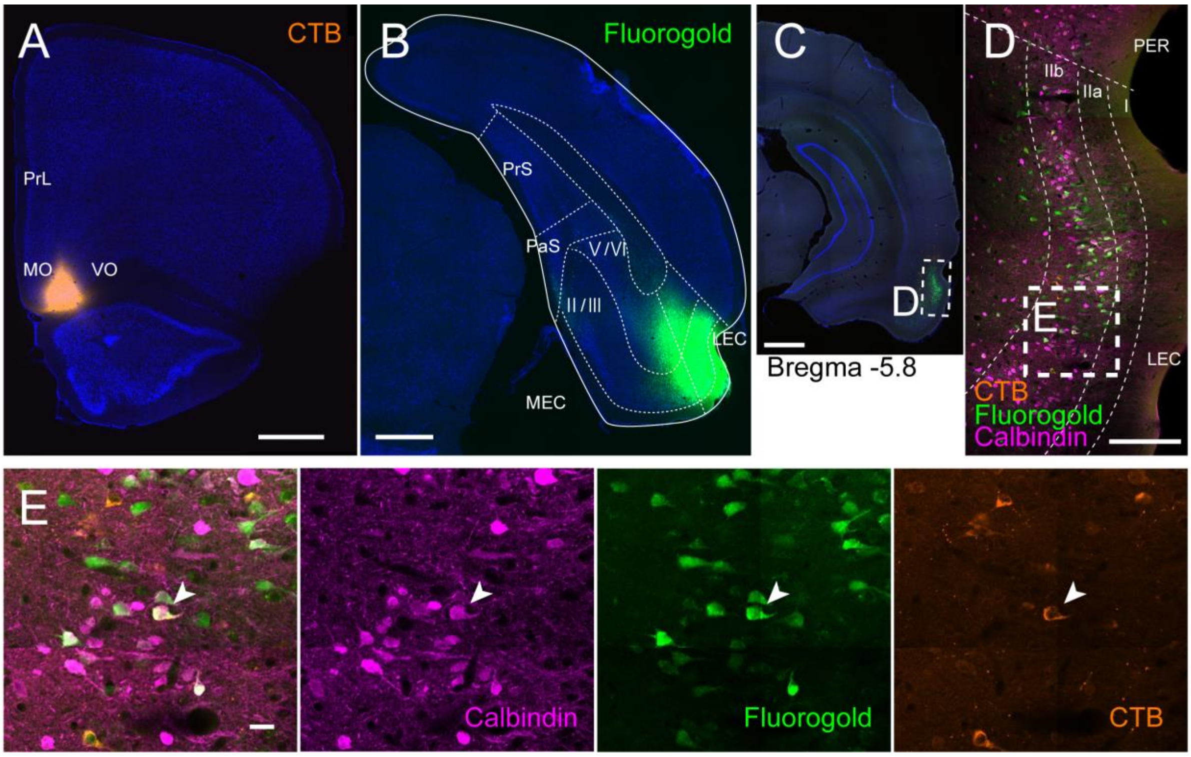

Supplementary Figure 7. Double labelling of retrogradely labelled neurons in LEC following an injection of CTB-555 in the ventral mPFC and FG in EC (A–B, case: EC1). (C–D) Distribution of retrogradely labeled neurons in ipsilateral LEC at an anteroposterior (AP) level of -5.8 mm in coronal sections. Boxed area in C is shown in D. (E) High magnification confocal images of the boxed area of LEC in (D). White arrows indicate neurons that were triple-labeled with FG, CTB-555, and CB immunoreactivity. Scale bars are 1000 µm for (A–C), 200 µm for (D), and 20 µm for (E).

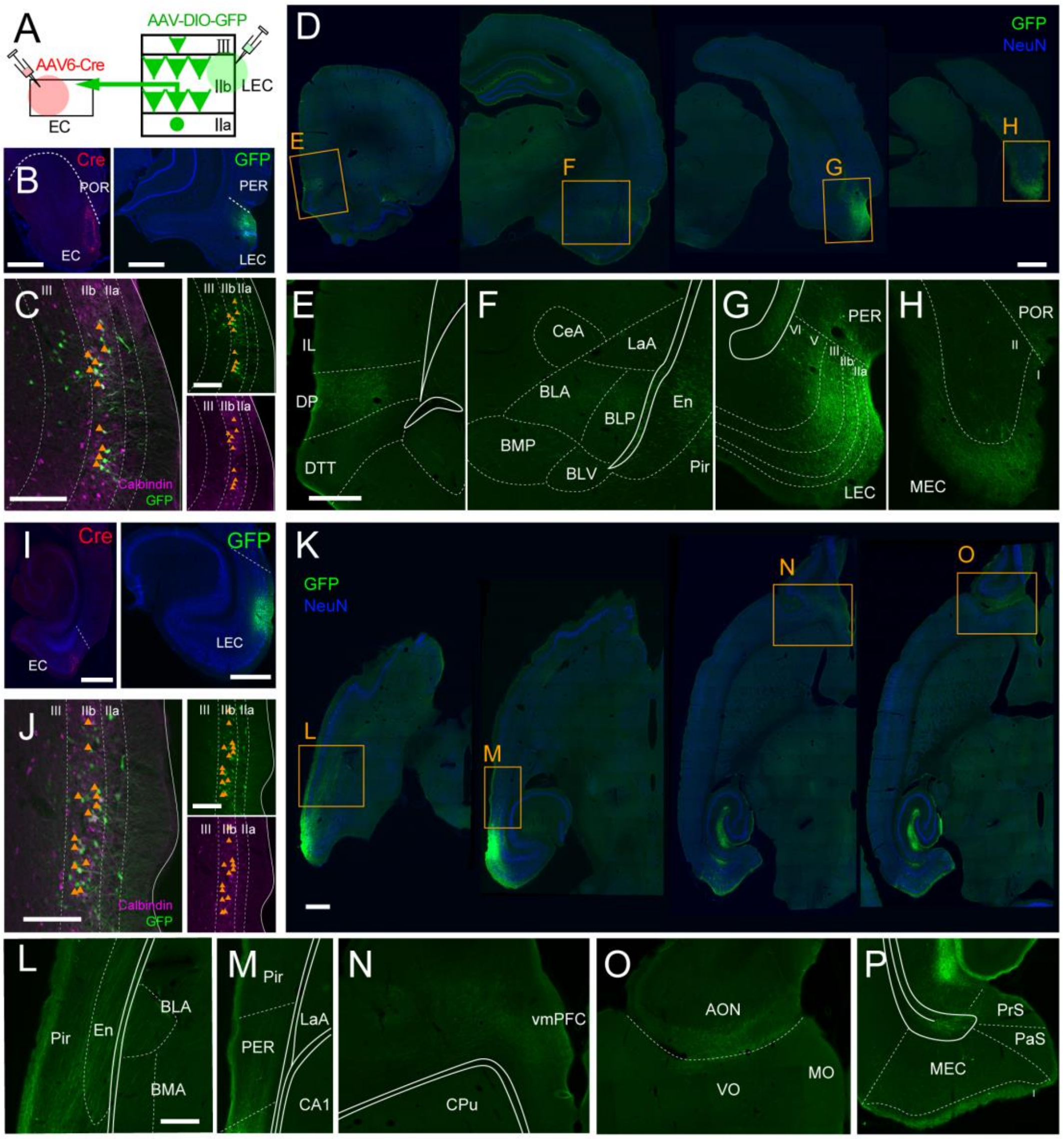

Supplementary Figure 8. Axon distribution of the local-projecting entorhinal neurons in the superficial layers. (A) Schematic diagram of the double infection approach to visualize local projecting LEC neurons in the superficial layers. (B, I) AAV6-Cre was injected in the border of LEC and MEC and AAV1/2-EF1α-DIO-EYFP was injected in LEC. The distributions of labeled fibers were examined either in coronal sections (D–H) or in horizontal sections (K–P). (C, J) High magnification image of the injection site of AAV1/2-EF1α-DIO-EYFP in LEC. Yellow arrows indicate double-labeled neurons with GFP (green) and CB (magenta). (D–H, K–O) Distribution of labeled fibers in low magnification images (D, K), and in high magnification images of boxed area in D (E–H) and in K (L–O). (P) High magnification image of MEC taken from a section ventral to the ones shown in K. Scale bars are 1000 µm for (B), (D), (I) and (K), 500 µm for (E) and (L), and 200 µm for (C) and (J). Abbreviations: BLA, basolateral amygdala; BLP, basolateral amygdala, posterior part; BLV, basolateral amygdala, ventral part; BMA, basomedial amygdala; BMP, basomedial amygdala, posterior part; CeA, central amygdala; CPu, caudate putatum; DP, dorsal peduncular cortex; DTT, dorsal tenia tecta; En, endopiriform nucleus; LaA, lateral amygdala; MO, medial orbital cortex; PaS, parasubiculum; PER, perirhinal cortex; POR, postrhinal cortex; PrS, presubiculum; VO, ventral orbital cortex.

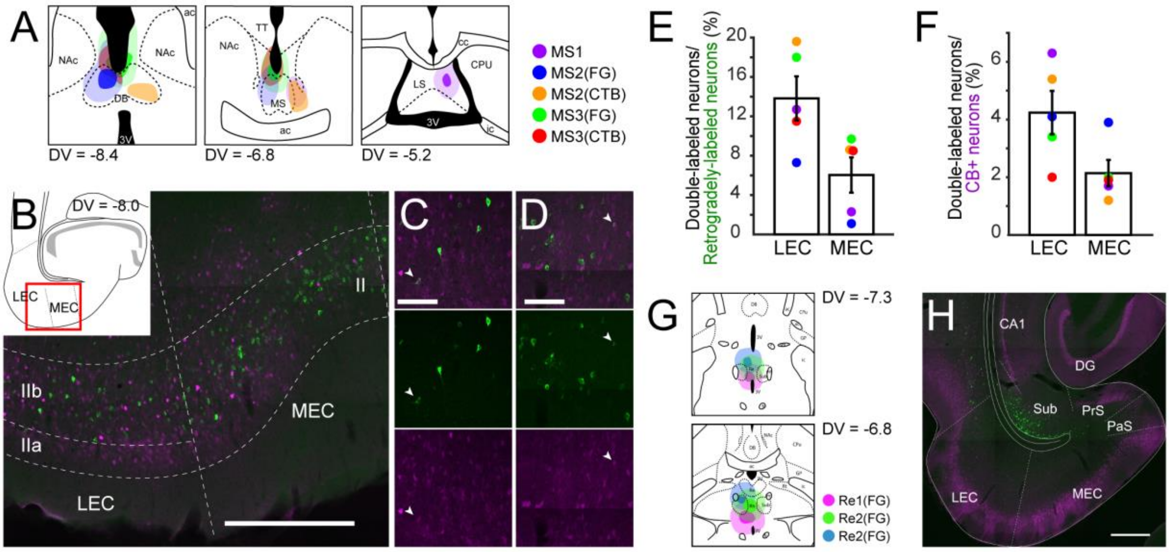

Supplementary Figure 9. Retrogradely-labeling in EC after retrograde tracer injection into septal complex (A–F) and thalamic nucleus reuniens (G–H). (A) The injection sites of retrograde tracers in samples with septal injection are shown in horizontal sections at three different dorsoventral level. Each injection is illustrated with a different color. The dark color shows the injection site and the light color shows the area of tracer diffusion. (B–D) Distribution of retrogradely labeled neurons (green) in ventral EC in horizontal section (case: MS2 (CTB)). High magnification images of the superficial layers in LEC and MEC are shown in (C) and (D) respectively. White arrows indicate neurons that were double-labeled with CTB-555 and CB immunoreactivity. (E, F) The percentage of double-labeled neurons among retrogradely-labeled neurons (E), and the percentage of double-labeled neurons among the CB+ neurons (F) are compared between LEC and MEC (mean ± standard errors, N = 5). (G) The injection sites of FG in samples with reuniens injection are shown in two horizontal sections at two different dorsoventral level. (H) Distribution of FG-labeled neurons in a horizontal section (case: Re1). Scale bars are 500 µm for (B) and (H), and 100 µm for (C) and (D).

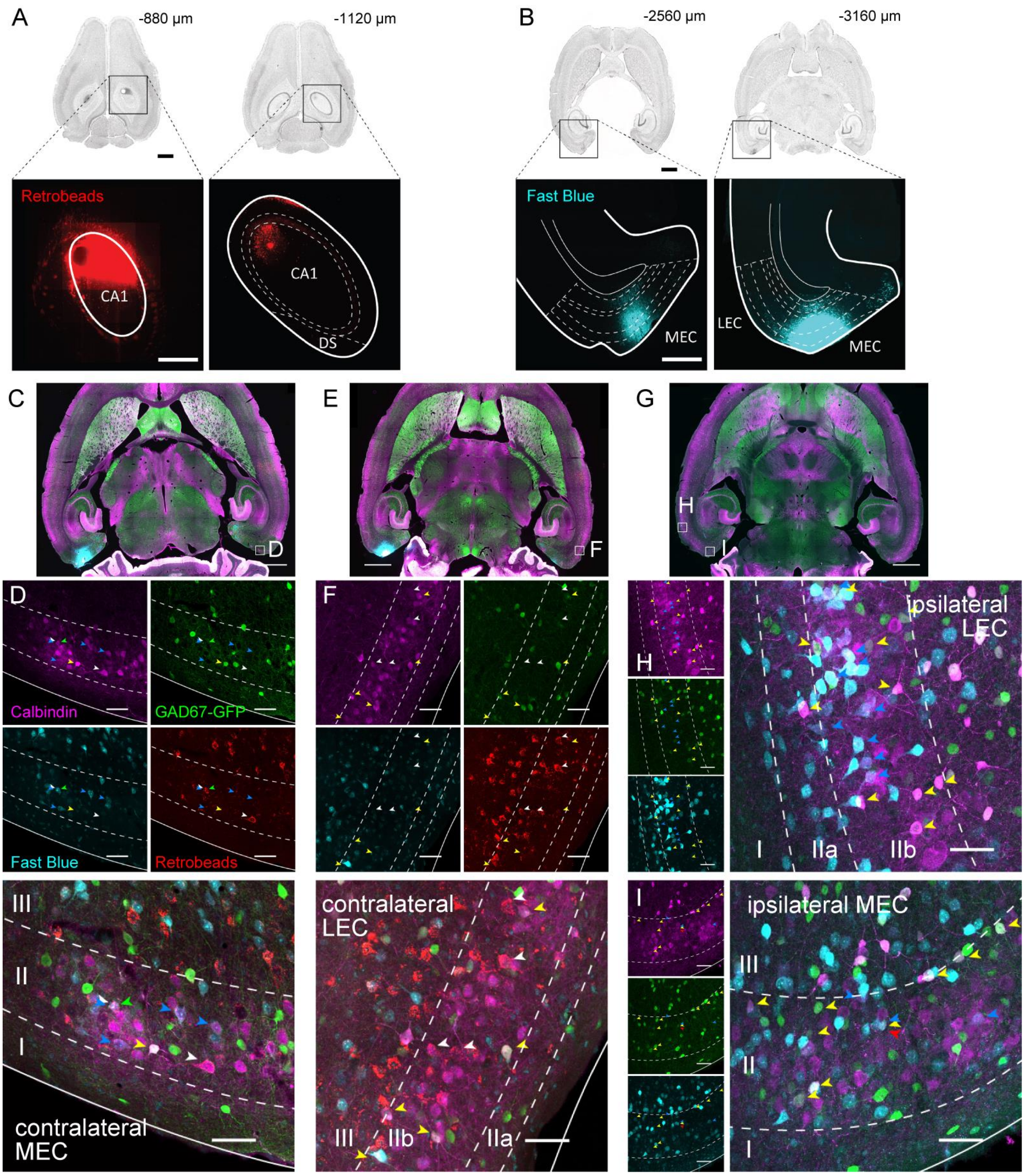

Supplementary Figure 10. Representative example of retrograde tracing in a GAD67 transgenic mouse expressing GFP, following injection of red retrobeads in dorsal CA1 (A), and Fast Blue in contralateral MEC (B). Colocalization between labeling for calbindin (magenta), GAD67-GFP (green), Fast Blue (cyan), and retrobeads (red) in MEC (C, D) and LEC (E, F) contralateral to Fast Blue injection, and LEC (G, H) and MEC (I) ipsilateral to fast blue injection. Retrogradely labeled CB+ positive neurons are common in LEC and MEC, both on the ipsilateral and contralateral side of the MEC injection. We rarely observed CB+/GAD+ retrogradely-labeled neurons and these were only present in MEC ipsilateral to the MEC injection (I). Blue arrows: Fast Blue labeled CB+ neurons. White arrows: retrobeads labeled CB+ neurons. Yellow arrows: GAD67-GFP expressing CB+ neurons. Green arrow: triple-labeled with Fast Blue, retrobeads, and CB (D). Red arrow: triple labeled with Fast Blue, GAD67-GFP, and CB (I). Scale bars are 1000 µm for (A–B black scale bars) and (C), (E), and (G), 500 µm for (A–B white bars), and 50 µm for (D, F, H, I).

Sample name	Species	sex	weight (g)	Injection location	Coordinates APb	Apt	ML	DV	Angle	Tracer	Volume (nl)	Cutting plane	Thickness (µm)	Reference in manuscript	Animal number
HIP1	W	M	200-230	CA1, CA3	-5.1		3.4	-2.3/-2.8		FG	200	H	60	Fig.1B-H, Sfig.3	j2039
HIP2	W	M	200-230	CA1, DG	-5.1		3.4	-2.4/-2.9		FG	200	H	60	Fig.1B,G,H, Sfig.3	j2040
HIP3	W	M	200-230	CA1, DG	-3.7		4.3	3		FG	100	H	60	Fig.1B,G,H, Sfig.3	j1989
HIP4	SD	F	200-230	CA1, DG	-3.6		2	-2.4		FB	150	H	40	Fig.1B,G,H, Sfig.3	20218
HIP5	SD	F	200-230	CA1	-3.6		2	-2.1		FB	150	H	40	Fig.1B,G,H, Sfig.3	20187
HIP6	SD	F	200-230	CA1	-3.6		2	-2.1		FB	150	H	40	Fig.1B,G,H, Sfig.3	20188
HIP7	SD	F	200-230	CA1	-3.6		2	-2.1		FB	150	H	40	Fig.1B,G,H, Sfig.3	20350
MEC1	LE	F	213	MEC		0.9	5.1	3.3		FB	40	H	40	Fig. 2A-G	24448
MEC2	LE	F	237	MEC		0.9	5.3	3.3		FB	40	H	40	Fig. 2A-G	24447
MEC3	LE	M	281	MEC		0.9	5.3	3.3		FB	40	H	40	Fig. 2A-G	24461
LEC1	LE	M	242	LEC, (PER)	-6.0		7.0	4.8		FG	50	H	40	Fig. 2H-N	24360
LEC2	LE	F	250	LEC, (PER)	-6.0		7.0	4.8		FG	50	H	40	Fig. 2H-N	24362
LEC3	LE	F	233	LEC, (PER)	-6.0		7.05	4.8		FG	75	H	40	Fig. 2H-N	24139
EC1	W	M	200-230	EC	-8.3		6.0	-4.0		FG	100	C	60	Sfig. 5A, F, G, Sfig. 7	j1994
				vmPFC	3.5		0.6	-4.2		CTB	60				
EC2	W	M	200-230	EC	-8.3		6.0	-4.0		FG	100	C	60	Sfig. 5A-G	j1995
EC3	W	M	200-230	EC	-8.3		6.0	-3.8		FG	100	C	60	Sfig. 5A, F, G	j1944
EC4	W	M	200-230	EC	-8.3		6.0	-4.0		FG	100	C	60	Sfig. 5A, F, G	j1993
EC5	W	M	200-230	left EC	-8.3		6.0	-4.0		FG	100	H	40	Sfig. 5A, F-H	j2547
				right EC	-8.3		6.0	-4.0		CTB	100				
EC6	W	M	200-230	left EC	-8.3		6.0	-4.0		FG	200	H	40	Sfig. 5A, F, G	j2548
				right EC	-8.3		6.0	-4.0		CTB	250				
OFC1	W	M	200-230	ventral OFC	3.7		2.2	-2.7		FG	180	C	60	Sfig. 6C	j1748
OFC2	W	M	200-230	ventral OFC	3.7		2.2	-3.7		FG	100	C	60		j1749
OFC3	W	M	200-230	ventral OFC	3.7		2.2	-3.2		FG	100	C	60		j1821
aPir1	W	M	200-230	anterior Pir	3.7		2.2	-5.5		FG	150	C	60	Sfig. 6B	j1822
aPir2	W	M	200-230	anterior Pir	3.7		2.3	-5.5		FG		C	60		j2464
dmPFC1	W	M	200-230	PrL	3.5		0.6	-2.6		FG	100	C	60	Sfig. 6A<br>Ohara et al., 2018	j1817
dmPFC2	W	M	200-230	PrL	3.5		0.6	-2.6		FG	100	C	60		j1818
AIC1	W	M	200-230	AIC	3.0		4.4	-4.2		FG	100	C	60		j1750
AIC2	W	M	200-230	AIC	3.0		4.4	-4.2		FG	80	C	60	Sfig. 6E	j1751
NAc1	W	M	200-230	NAc	2.5		1.6	-6.1		FG	120	C	60	Ohara et al., 2018	j1833
NAc2	W	M	200-230	NAc	2.5		1.6	-6.1		FG	100	C	60	Sfig. 6D	j1834
RSC1	W	M	200-230	RSC	-7.0		0.9	1.2		FG	50	C	60		j2334
RSC2	W	M	200-230	RSC	-7.0		0.9	1.2		FG	60	C	60	Sfig. 6F<br>Ohara et al., 2018	j2335
RSC3	W	M	200-230	RSC	-7.7		0.8	1.5		FG	100	S	60		j2465
POR1	SD	F	270g	left POR		0.5	4.4	1.4		FG	50	H	40	Sfig. 6G	24585
				right POR		0.5	4.4	1.4		CTB	500				
vmPFC1	W	M	200-230	IL, DP, MO	3.5		0.6	4.2		FG	100	C	60	Fig. 3D	j1773
vmPFC2	W	M	200-230	IL, DP, MO	3.5		0.6	4.2		FG	125	C	60	Fig. 3D-F, Sfig. 6H	j1795
AMG1	W	M	200-230	AMG	-2.3		5.1	7.0		FG	100	C	60	Fig. 3A-C, Sfig. 6I<br>Ohara et al., 2018	j1736
AMG2	W	M	200-230	AMG	-2.3		5.1	7.0		FG	100	C	60	Fig. 3A	j1737
AON1	SD	F	250	AON	5.5		1.2	-4.2		FG	150	C	40	Fig. 3J	25042
AON2	SD	F	280	AON	5.5		1.2	-4.2		FG	100	C	40	Fig. 3J-L, Sfig. 6J	25043
pPir1	SD	F	240	posterior Pir	-2.7		6.0	-6.7		FG	80	C	40	Fig. 3G	25040
pPir2	SD	F	240	posterior Pir	-2.7		6.1	-6.7		FG	100	C	40	Fig. 3G-I, Sfig. 6K	25041
PER1	SD	F	300	PER	-5.0		6.8	-4.5		FG	100	H	40	Fig. 3M	24801
PER2	SD	F	260	PER	-5.0		6.8	-4.5		FG	100	H	40	Fig. 3M-O, Sfig. 6L	25044
MS1	W	M	200-230	LS	0.5		0.6	-5.0		FG	120	H	40	Sfig. 9A, E-F	j2471
MS2	W	M	200-230	right MS/DB	0.5		0.6	-4.2/-4.0		FG	250	H	40	Sfig. 9A-F	j2549
	W			left MS/DB	-0.1		0.6	-4.0/-3.8		CTB	300				
MS3	W	M	200-230	left MS/DB	-0.1		0.6	-4.0/-3.8		FG	250	H	40	Sfig. 9A, E-F	j2550
	W			right MS/DB	0.5		0.6	-4.2/-4.0		CTB	200				
Re1	SD	F	250	Re	-2.5		2.8	7.3	22	FG	200	H	40	Sfig. 9G, H	24128
Re2	SD	F	240	Re	-2.2		2.8	7.3	22	FG	125	H	40	Sfig. 9G	24420
Re3	SD	F	230	Re	-2.3		2.8	7.3	22	FG	125	H	40	Sfig. 9G	24424

Supplementary Table 1. Injection parameters of retrograde tracing experiments. Either the Fluorogold (FG), Fast Blue (FB), or Alexa Fluor 555 conjugated Cholera Toxin Subunit B (CTB) was used. In the Coordinates column, APb stands for anterior to bregma, APt for anterior to transverse sinus, ML for lateral to sagittal sinus, and DV for ventral to dura. These coordinates are shown in mm. Angle shows the degree angle of the injection micropipette in the coronal plane with the tip pointing to the midline. Long Evans (LE), Sprague Dawley (SD), Wistar (W). Male (M) and Female (F). Horizontal plane (H), Coronal plane (C), Sagittal plane (S).
